# Microbial phylogeny determines transcriptional response of resistome to dynamic composting processes

**DOI:** 10.1186/s40168-017-0324-0

**Published:** 2017-08-16

**Authors:** Cheng Wang, Da Dong, P. J. Strong, Weijing Zhu, Zhuang Ma, Yong Qin, Weixiang Wu

**Affiliations:** 1 Zhejiang Province Key Laboratory for Water Pollution Control and Environmental Safety Technology, Institute of Environmental Science and Technology, Zhejiang University, 866 Yuhangtang Road, Hangzhou, 310058 China; 2 Zhejiang Provincial Key Laboratory of Carbon Cycling in Forest Ecosystems and Carbon Sequestration, Zhejiang A & F University, Lin’an, 311300 China; 3 Queensland University of Technology, GPO Box 2432, 2 George St, Brisbane, QLD 4001 Australia

**Keywords:** Resistome response, Metatranscriptomics, Metagenomics, Composting, ARGs, MGEs

## Abstract

**Background:**

Animal manure is a reservoir of antibiotic resistance genes (ARGs) that pose a potential health risk globally, especially for resistance to the antibiotics commonly used in livestock production (such as tetracycline, sulfonamide, and fluoroquinolone). Currently, the effects of biological treatment (composting) on the transcriptional response of manure ARGs and their microbial hosts are not well characterized. Composting is a dynamic process that consists of four distinct phases that are distinguished by the temperature resulting from microbial activity, namely the mesophilic, thermophilic, cooling, and maturing phases. In this study, changes of resistome expression were determined and related to active microbiome profiles during the dynamic composting process. This was achieved by integrating metagenomic and time series metatranscriptomic data for the evolving microbial community during composting.

**Results:**

Composting noticeably reduced the aggregated expression level of the manure resistome, which primarily consisted of genes encoding for tetracycline, vancomycin, fluoroquinolone, beta-lactam, and aminoglycoside resistance, as well as efflux pumps. Furthermore, a varied transcriptional response of resistome to composting at the ARG levels was highlighted. The expression of tetracycline resistance genes (*tetM*-*tetW*-*tetO*-*tetS*) decreased during composting, where distinctive shifts in the four phases of composting were related to variations in antibiotic concentration. Composting had no effect on the expression of sulfonamide and fluoroquinolone resistance genes, which increased slightly during the thermophilic phase and then decreased to initial levels. As indigenous populations switched greatly throughout the dynamic composting, the core resistome persisted and their reservoir hosts’ composition was significantly correlated with dynamic active microbial phylogenetic structure. Hosts for sulfonamide and fuoroquinolone resistance genes changed notably in phylognetic structure and underwent an initial increase and then a decrease in abundance. By contrast, hosts for tetracycline resistance genes (*tetM*-*tetW*-*tetO*-*tetS*) exhibited a constant decline through time.

**Conclusions:**

The transcriptional patterns of a core resistome over the course of composting were identified, and microbial phylogeny was the key determinant in defining the varied transcriptional response of resistome to this dynamic biological process. This research demonstrated the benefits of composting for manure treatment. It reduced the risk of emerging environmental contaminants such as tetracyclines, tetracycline resistance genes, and clinically relevant pathogens carrying ARGs, as well as RNA viruses and bacteriophages.

**Electronic supplementary material:**

The online version of this article (doi:10.1186/s40168-017-0324-0) contains supplementary material, which is available to authorized users.

## Background

Although antibiotics have revolutionized modern medicine, microbial resistance has diminished their efficacy and is of global concern for public health. One of the greatest global consumers of antibiotics is livestock farming. In 1950, scientists from the USA observed that adding antibiotics to animal feed increased the growth rate of livestock [1]. This practice was so effective that 50 years later, more than 80% of the antibiotics consumed in the USA was for non-therapeutic agricultural production [2]. This practice was also adopted in China, and currently, almost 50% of all antibiotics produced in China are used in animal husbandry for prophylactic needs and growth promotion [3]. The widespread use of antibiotics contributes to the emergence and dissemination of resistant bacteria in the environment, which include pathogenic human bacteria [4, 5]. Early studies circumstantially indicated that livestock manure was a “hot spot” for bacteria carrying a diverse set of antibiotic resistance genes (ARGs) [6, 7]. The direct disposal of manure introduced not only antibiotics but also considerable numbers of bacteria carrying ARGs into the receiving environments [8, 9]. Disposing of raw manure could promote the transfer of genes for antibiotic resistance [10]. As a consequence, livestock manure requires pretreatment to prevent the environmental contamination with antibiotic-resistant bacteria and the transmission of their ARGs.

Composting is an established sustainable practice where organic waste (such as livestock manure) is aerobically degraded. It produces a marketable organic fertilizer as a residue [11]. Composting is an aerobic and self-heating bioprocess with localized temperature, pH, oxygen, moisture, and nutrient gradients, which create highly heterogeneous microniches of distinctly adapted microbial populations [12]. Although composting does degrade antibiotics and reduce ARGs [13, 14], most studies have focused on a few well-studied types of ARGs such as tetracycline and sulfonamide resistance genes. The lack of comprehensive antibiotic resistance studies, therefore, provide a strong impetus to better understand the genetic context and fate of the complete resistome (comprising all of the antibiotic resistance genes and their precursors [15]) during composting, as well as critically evaluate the potential of this biological treatment to attenuate antibiotic resistance determinants and limit their dissemination. Given the observation of resistomes structured by microbial communities [16, 17], composting may be regarded as a platform to systematically investigate the response of a resistome to shifts in indigenous microbial community structure during a dynamic biological treatment process.

Recent advances in molecular biology have enabled the assessment of the resistance gene load of a given environment’s microbial community. Several studies of soil and human habitats [16, 18] have demonstrated the power of selections in the functional analysis of metagenomic libraries, with a great advantage in qualifying ARGs and resistance phenotypes under antibiotic stress. Although such studies provide comprehensive information on the diversity and abundance of putative ARGs, knowledge concerning the microbial traits that actually contribute to antibiotic resistance is scarce. With the increasing detection of ARGs in environments without anthropogenic effects [19, 20], metatranscriptomic analysis is urgently required to determine whether those potentially functional genes predicted from metagenomes were partially or fully expressed. Furthermore, considering that the dissemination of antibiotic resistance determinants in a bacterial population is frequently regulated by environmental and genetic factors [21], the integration of metagenomic and metatranscriptomic data is expected to yield a deeper insight into the antibiotic resistomes, genetic factors involved, and their expression profiles within the compost ecosystem. The comparison of the temporal dynamics of transcriptional resistome over composting allows better understanding the mechanisms that drive the prevalence and dissemination of ARG transcripts under changing environmental conditions.

In this study, we integrated metagenomic and metatranscriptomic data to systematically explore the resistome profiles in compost ecosystems. The metatranscriptomic analysis leveraged a four-phase (mesophilic, thermophilic, cooling, and maturing phases) time series that spanned the entire composting process, where each phase was distinguished by temperature (Additional file 1: Figure S1). Samples in each of the four typical phases are environmentally distinct [22]. Raw materials from the mesophilic phase had the highest moisture content. A high temperature characterized the thermophilic phase. During the cooling phase, the temperature declined with a lower moisture content, finally shifting to the maturing phase. Whole community-scale metatranscriptomes from compost microbiota inhabiting these distinct phases offer a unique opportunity to investigate the dynamics of transcriptional resistome and its putative host under rapidly changing environmental conditions. Furthermore, a metagenomic analysis was performed to explore the resistome profile of a microbial consortia developed from the compost habitat. By integrating the metagenomic and metatranscriptomic data, we evaluate the extent to which putative resistome genes in compost microbiome were expressed. With the aim of providing reliable and comparable results on active microbiome profiling and resistome dynamics during composting, two compost piles with different kinetics were conducted at a pilot scale (2 t each) in this study. To explore factors that influence the emergence and spread of antibiotic resistance during composting, the correlation between the concentration of primary antibiotics (tetracycline, sulfonamide, and fluoroquinolone) and their corresponding ARG abundance and transcriptional activity were analyzed in both pilot-scale piles. The expression profiles of mobile genetic elements (MGEs) that play a vital role in antibiotic resistance transmission were also examined. Ultimately, these results provide deeper insights into microbial mechanisms of antibiotic resistance dissemination during composting, which is essential for assessing the risks posed by the environmental release of agricultural antibiotics, ARGs, and ARG-carrying microorganisms.

## Methods

### Composting experiments and sampling

Aerobic windrow compost piles of pig manure (2 t each) were constructed in a suburb of Hangzhou in China and monitored for approximately 12 weeks. The piles consisted of pig manure and a bulking agent (wood chips and sawdust). The pig manure was collected from the pig production facilities within the local area. The wood chips and sawdust were milled to yield a 5-cm maximum particle size, and then homogeneously mixed with pig manure. In this study, we conducted two different pilot-scale treatments. The two treatments were referred as PWS (1200 kg pig manure, 400 kg wood chips, and 400 kg sawdust) or PWSB (1200 kg pig manure, 400 kg wood chips, 400 kg sawdust, and 60 kg biochar). Our previous studies have reported that amendment of biochar, a well-defined environmentally friendly material [23], was able to enhance the kinetics of composting [22]. The moisture content of the material was initially adjusted to approximately 65%, and there were no further adjustments throughout the whole composting period. Aeration was via natural ventilation and turning. Both compost piles were periodically remixed and turned over with a wheel loader.

On days 3, 22, 48, and 61 of composting, 500 g subsamples were removed from six sites of the entire profile (horizontal and vertical sampling) and mixed together to yield one composite sample. The composite samples were divided into two parts that were both tested in triplicate for nucleic acid extraction and antibiotics analyses. One part was immediately stored at −20 °C until analyses, while the other was freeze-dried using a FreeZone freeze dry system (Labconco, Kansas City, MO), homogenized by sieving through a 0.15-mm mesh, and stored in a desiccator prior to antibiotics analyses. The temperatures at 20 cm depth below the surface of the compost piles and ambient air were recorded daily to qualify the performance of the composting process. Based on the composting temperature [22], samples on days 3, 22, 48, and 61 of composting were analyzed as they were representatives of the mesophilic, thermophilic, cooling, and maturing phases of composting, respectively.

### Nucleic acid extraction and metatranscriptomic and metagenomic library preparation and sequencing

Three independent RNA extracts were extracted from 0.5 g compost samples from both PWS and PWSB piles using a E.Z.N.A.® Soil RNA Kit (Omega BioTek, Inc., Norcross, GA, USA) according to the manufacturer’s instructions. Extracts were then added with DNase I (TaKara, China), by incubation at 37 °C for 1 h to eliminate traces of DNA. The RNA quality was determined using Agilent 2100 bioanalyzer and quantified using the NanoDrop (Thermo Scientific, Wilmington, DE, USA). Ribosomal RNA was removed from the total RNA samples using an Epicentre Ribo-zero™ rRNA Removal Kit (Epicentre, Madison, WI, USA). Prior to the construction of libraries using the TruSeqTM RNA Sample Preparation Kits v2 (Illumina®), three independent RNA extracts of each sample were combined. The pooled libraries were sequenced on an Illumina MiSeq platform (Majorbio, Shanghai) according to standard protocols to generate more than 33 million reads, averaging 356 nt per sample (Additional file 2: Table S1).

In our previous study, a microbial consortia (RSA consortia) adherent to rice straw was developed from the compost habitat, and its metagenomic DNA was sequenced [24]. Here, we analyzed the metagenomic dataset from this typical compost microbiome to systematically investigate the resistome profile. By comparing the metagenomic to the metatranscriptomic data, we endeavored to evaluate the extent to which putative resistome genes predicted from metagenomes were expressed. As previously described [24], high molecular weight DNA was extracted using the MoBio UltraClean Soil DNA isolation kit (MoBio Laboratories, Solana Beach, CA, USA) according to the manufacturer’s instructions. The concentrations and quality of DNA samples were measured using the NanoDrop (Thermo Scientific, Wilmington, DE, USA). The DNA was eluted in a final volume of 100 μl and the eluents were stored at −20 °C until further quantification analysis and metagenomic sequencing. Metagenomic library preparation and sequencing were performed by BGI (Shenzhen, China) with the Illumina Genome Analyzer technology, and followed BGI’s previous work on human gut microbiome metagenomic sequencing [25]. A library with 180-bp clone insert size was constructed and nearly 59.6 million high-quality reads were generated.

### Metagenomic and metatranscriptomic sequence analysis

Raw metatranscriptomic reads were quality-trimmed after adaptor and contaminant removal using bbduk tool in BBMap (V34: https://sourceforge.net/projects/bbmap/; minimum quality value of 20; minimum read length ≥ 50 bp). In each complementary DNA (cDNA) data set, non-ribosomal RNA (rRNA) sequences were checked for replicate sequences using the open-source program CD-HIT [26]. Replicates were defined as sequences sharing greater than 99% nucleotide identity, with an allowable length difference of 1 bp and a requirement that the first 3 bp of the replicate sequences should be identical. The resulting non-rRNA, non-replicate cDNA sequences were searched for in the NCBI non-redundant database, and the results were parsed using the lowest common ancestor algorithm in MEGAN [27] to obtain their taxonomic information [28]. Metagenomic sequence reads were processed and analyzed as described previously [24]. Briefly, all of the metagenomic sequence reads were quality-trimmed to an accuracy of 99.4%, and duplicate reads were identified and removed prior to assembly. High-quality short reads of the DNA sample were assembled by the SOAPdenovo assembler [29], as described in human gut microbiome metagenomic analyses [30]. After assessing different Kmer sizes, we used the contigs of Kmer 41 for our sample as the final assembly result. ORFs were predicted by using the gene-finding algorithm MetaGeneMark [31].

The abundance and diversity of ARG families in the metatranscriptomic and metagenomic raw sequence data sets were analyzed by screening the antibiotic resistance protein families with the HMMER package (version 3.1b1) [32] using a collection of custom-built, resistance-gene-specific profiles HMMs (*e* value 10^− 5^, http://www.dantaslab.org/resfams/) [33]. The detected antibiotic resistance genes and transcripts were screened (BLASTP) against the NCBI non-redundant database (NCBInr) [34] to predict their phylogenetic origin. The minimum query coverage and sequence identity were both 70%, with a threshold *e* value of 10^− 5^. Putative MGEs and their transcripts were searched for and screened on the PFAM and TIGRFAMS databases [35, 36], with the same data-processing workflow to soil microbiomes [37]. To further assess potential ARG mobility, the assembled metagenomic contigs were also aligned to plasmid genome sequences available in the NCBI RefSeq database, and this alignment was performed against the known plasmids using BLAST. BLAST hits (blastn) were determined with a nucleotide identity above 95% over a length of at least 90 bp (*e* value 10^− 10^). Antibiotic-resistant ORFs were considered co-localized with an MGE if they shared a contig with a MGE ORF. The relative abundance of a given taxon in a community was calculated as the percentage of the number of sequences assigned to the taxon divided by the total number of sequences assigned to all the taxa in the community. Similar calculations were performed for relative abundance of a given ARG type. When visualizing the distribution profiles of ARG-carrying hosts at the species level, the abundance values were natural logarithm-transformed to normalize their distribution. The DNA nucleotide sequences are deposited at MG-RAST under the accession numbers of 4513787.3, and the cDNA nucleotide sequences are deposited at MG-RAST under the accession numbers of mgs491092, mgs491095, mgs491098, mgs491101, mgs491104, mgs491107, mgs491110, and mgs491113 (http://metagenomics.anl.gov/mgmain.html?mgpage=project&project=mgp18601).

### Quantification of antibiotics

In this study, we investigated a total of 12 antibiotics belonging to the classes of tetracycline (tetracycline (TC), chlortetracycline (CTC), oxytetracycline (OTC), and doxycycline (DOC)), sulfonamide (sulfadiazine (SDZ), sulfamerazine (SMZ), sulfamethoxazole (SMX), and sulfaclozine (SCZ)), and fluoroquinolone (norfloxacin (NFC), ciprofloxacin (CFC), ofloxacin (OFC), and enrofloxacin (EFC)) that were widely used in livestock industries [38]. The concentrations of antibiotics were determined by liquid chromatography-tandem mass spectrometry with the isotope-labeled internal standard method as previously described [39]. Briefly, each dried and homogenized sample (0.2 g) was spiked with the surrogate standards and internal standards (100.0 μg kg^− 1^), which was followed by adding the extraction solvent consisting of EDTA-SPB with acetonitrile (Mg(NO_3_)_2_-NH_3_·H_2_O, *v*/*v*, 3:1). The mixtures were placed in the dark overnight (12–14 h). The samples were shaken for 30 min at 200 rpm in the dark the following day, sonicated for 15 min, and centrifuged at 5000 rpm for 10 min. The supernatant was then collected. The extraction protocol was repeated three times per sample. The combined supernatants were filtered through a 0.7-μm glass microfiber filter (GF/F, Whatman) and diluted to 500 ml with ultrapure water to maintain an organic solvent content ≤ 5% in the extract. Next, we extracted the antibiotics using ultrasonic-assisted extraction, which was followed by solid phase extraction clean-up with hydrophilic-lipophilic balance cartridges. Finally, the antibiotics were separated and detected using liquid chromatography-electrospray ionization tandem mass spectrometry (LC-ESI-MS/MS) and quantified using the isotope-labeled internal standard method. Mass spectral acquisition was performed in the positive ion mode by applying multiple reactions that monitored two fragmentation transitions per compound—to provide a high degree of sensitivity and specificity.

### Real-time quantitative PCR (q-PCR) of ARGs

Four tetracycline resistance genes (*tetM*, *tetW*, *tetO*, and *tetQ*), two sulfonamide resistance genes (*sulI* and *sulII*), and the integrase gene of class 1 integrons (*intI1*) were quantified using q-PCR. The q-PCR procedure was performed as previously described in Chen et al. [40]. Target genes in positive controls (including four *tet* genes, two *sul* genes, and class 1 integrons) were obtained from compost DNA extracts after PCR amplification and then cloned into *Escherichia coli* DH5a (TaKaRa). After verifying cloned target genes and sequencing, clones containing the target gene inserts served as standards for q-PCR. The quantification was based on the intensity of SYBR Green dye fluorescence, which bound to double-stranded DNA. The PCR reaction mixture consisted of 0.2 μM of each primer, 7.5 μl of SYBR Premix Ex Taq™ (TaKaRa), 0.3 μl of ROX reference dye, 2 μl of template DNA, and 4.6 μl of ddH_2_O. A StepOne Plus™ real-time PCR system (ABI, USA) was used as follows: 30 s at 95 °C, then 40 cycles of 5 s at 95 °C, 30 s at the annealing temperature, extension for another 30 s at 72 °C and monitored with simultaneous fluorescence signal scanning at 72 °C, then a melt curve stage with temperature ramping from 60 to 95 °C. All real-time PCR assays were performed using three replicates per sample, and all PCR runs included control reactions without the template. Standard curves were obtained using serial dilutions of linearized plasmids containing target genes. Based on the standard curves, the *C*_t_ value of unknown samples was used to calculate the copy number of corresponding genes. The *R* ^2^ values of all the standard curves were greater than 0.98, and the calculated PCR efficiencies were 89.8 to 105.3%. The q-PCR primers for the target genes and the annealing temperatures used are shown in Additional file 3: Table S2.

### Statistics

Redundancy analysis between environmental parameters and microbial communities, Procrustes test for correlation analysis between ARGs and microbial communities, and heatmap were performed in R3.1.0 (https://www.r-project.org/) with vegan [41] and a pheatmap package [42]. Spearman correlation, Kruskal-Wallis, and Tukey’s tests were performed using SPSS V20.0 (IBM, USA).

## Results and discussion

### Transcriptional response of ARG types to composting

**Fig. 1:**
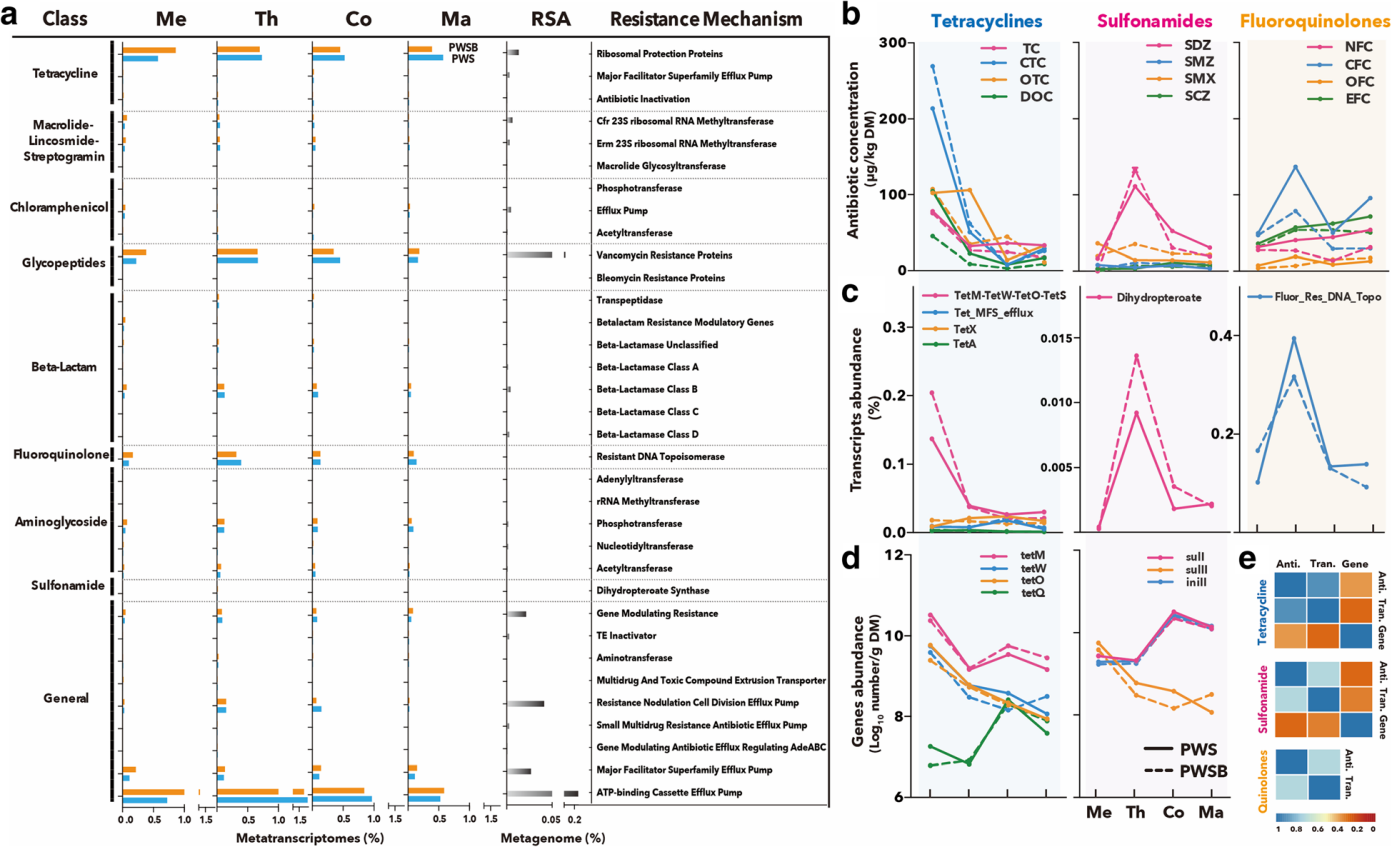
Changes in ARG transcripts, ARG types, and antibiotics during the whole composting process. **a** Expression dynamics of resistome in our metatranscriptoms of microbiome covering four typical phases of the whole composting process in PWS and PWSB, and resistome profile in our metagenome of a unique rice straw-adapted (RSA) consortia enriched from the compost habitat; resistome data are shown at the resistance mechanism level, and “Me,” “Th,” “Co,” and “Ma” represent the mesophilic, thermophilic, cooling, and maturing phases, respectively. **b** Changes in the concentration of tetracyclines (tetracycline (TC), chlortetracycline (CTC), oxytetracycline (OTC), and doxycycline (DOC)), sulfonamides (sulfadiazine (SDZ), sulfamerazine (SMZ), sulfamethoxazole (SMX), and sulfaclozine (SCZ)), and fluoroquinolones (norfloxacin (NFC), ciprofloxacin (CFC), ofloxacin (OFC), and enrofloxacin (EFC)) during the whole composting process. **c** Changes in the expression level of tetracycline, sulfonamide, and fluoroquinolone resistance genes during the whole composting process. **d** Changes in the abundance of tetracycline, sulfonamide, and fluoroquinolone resistance genes during the whole composting process. **e** Correlation matrices between the concentration of antibiotic compounds, the abundance of ARGs, and their expression level in the tetracycline, sulfonamide, and fluoroquinolone resistance; *orange boxes* represent low-correlation coefficients, whereas *blue boxes* represent stronger correlations

To profile the resistome in the compost ecosystem, the reservoir of ARGs associated with compost microbiota was characterized by metagenomic and metatranscriptomic data. One hundred and seventy-one unique ARG types were detected (as annotated using Resfams). Reads aligning to genes that encoded resistance to tetracycline, vancomycin, fluoroquinolone, beta-lactam, and aminoglycoside were extensively distributed and predominantly expressed. The key determinants of the resistome in the compost ecosystem were tetracycline resistance ribosomal protection protein, *vanR*, resistant DNA topoisomerase, lactamase B, and APH(3″) (Additional file 4: Figure S2). These predominant determinants, combined with efflux pumps (*msbA*, *drrA*, *macB*, and *macA* for the ATP-binding cassette efflux pump; MFS-1 and *emrB* for major facilitator superfamily efflux pump), accounted for over 85% of the total ARG abundance and represented the core antibiotic resistome in the compost ecosystem. Importantly, the expression pattern of the core antibiotic resistome in the compost ecosystem not only mirrored the resistome profile in our metagenome of an enriched consortia derived from compost habitats (Fig. 1a) but also matched with the widespread occurrence of the tetracycline, vancomycin, aminoglycoside, beta-lactam, and macrolide-lincosmide-streptogramin (MLS) resistance genes detected in feces from livestock farms [43]. This consistency indicates that the majority of the feces-specific ARG types were highly expressed in the compost ecosystem.

In order to assess the expression dynamics of resistome over the entire composting process, we compared the resistome profiles of samples from mesophilic, thermophilic, cooling, and maturing phase of composting in PWS and PWSB. The non-metric multidimensional scaling (NMDS) ordination at the resistance class, mechanism, and ARG levels using Euclidean distances revealed that there was no significant shift in expression profiles of the resistome during the entire composting (Additional file 5: Figure S3). However, the relative abundance of expressed resistome varied over the entire period at the resistance mechanism level (Fig. 1a). It increased significantly during the active phase of composting and decreased during the cooling and maturing stages. Especially for the three most prevalent resistance mechanisms (ATP-binding cassette antibiotic efflux pumps, tetracycline resistance, and vancomycin resistance proteins), the matured compost sample had the lowest abundance among the four samples (Fig. 1a), with 17% less abundance in the PWS and 55% less abundance in the PWSB. The data for both treatment procedures reveal that a noticeable reduction of the aggregated level of expression of ARGs by composting and that a biochar amendment likely improved the reduction.

The abundance of expressed resistome showed statistical differences at the ARG levels (*P* = 0.008, Kruskal-Wallis for multiple comparisons), and ARG types exhibited varied response to composting (Additional file 6: Figure S4). For example, genes encoding resistance to sulfonamide (dihydropteroate synthase), fluoroquinolone (DNA topoisomerase), glycopeptides (*vanR*, *vanS*, and *vanY*), aminoglycoside (APH(3″), AAC(6′)-I), chloramphenicol (CAT), beta-lactam (*cblA*), and efflux pumps (*msbA*, *drrA*, *macB*, *macA*, MATE, and RND efflux pumps) were most highly expressed during the thermophilic phase (*P* = 0.009, Kruskal-Wallis). In addition, many ARG types were initially highly expressed, but their abundance significantly declined in the maturing phase (e.g., *tetM*-*tetW*-*tetO*-*tetS*, *emrB*, *balI*, Usp, *ermA*, *ermB*, *ermC*, APH(3), ANT(6), *mprF*, and chloramphenicol phosphotransferase) (*P* < 0.001, Kruskal-Wallis). Conversely, a few ARG types (primarily involved in gene modulation and multidrug efflux: *soxR*, APH, *romA*, and *mexC*) displayed an elevated expression in the maturing phase (*P* = 0.037, Kruskal-Wallis). It was concluded that the transcriptional response of the resistome to composting varied at the ARG levels. This is consistent with the literature, where ARGs have differed in response to biological treatment [44].

To further elucidate factors affecting transcriptional responses of ARGs, the abundance of resistance genes and transcripts and the corresponding compounds’ concentrations were determined for tetracyclines (TCs), sulfonamides (SAs), and fluoroquinolones (FQs). They represent the primary antibiotics commonly used in the livestock industry [38]. Changes in the 12 compounds from these three antibiotics classes during the composting process are displayed in Fig. 1b. In raw manure, TCs were the dominant antibiotics (45.3 to 269.0 μg kg^− 1^), while FQs (4.0 to 49.5 μg kg^− 1^) and SAs (2.9 to 36.2 μg kg^− 1^) were less abundant. Interestingly, upon entering the thermophilic phase, there was a decrease trend in all TCs’ concentrations, while SDZ in SAs and CFC in FQs showed the opposite trend. In the subsequent cooling phase, the concentrations of SDZ and CFC were similar to initial values. Composting eliminated 77.6 to 87.3% of TCs but did not lower the concentrations of FQs and SAs (Fig. 1b). The different response of TCs, SAs, and FQs to thermophilic composting raises the possibility that temperature (which is a function of microbial activity) influenced the bioavailability and transformation of antibiotics [45]. Importantly, the greatest change in TCs, SAs, and FQs concentrations during the active phase of composting corresponded to substantial variations in the abundance of the corresponding resistance gene transcripts (Fig. 1c). The expression of sulfonamide and fluoroquinolone resistance genes increased in the thermophilic phase and then decreased to initial levels, whereas the tetracycline resistance genes (*tetM*-*tetW*-*tetO*-*tetS*) expression decreased throughout. Spearman rank correlations matrix revealed that antibiotic concentrations correlated with the corresponding ARG transcript abundance (correlation coefficient rho = 0.714 to 0.905, *P* < 0.05, Fig. 1e), suggesting that the antibiotic concentration influences the transcription profile of ARG types. Additionally, the distinct response of ARG types is also supported by the variations in ARGs abundance detected by q-PCR (Fig. 1d). A remarkable reduction of *tetM*, *tetW*, *tetO*, and *sulII* was observed (Tukey’s test, *P* < 0.01), while *tetQ* and *sulI* generally increased (*P* < 0.05). Although the ARG abundances displayed no significant correlations to ARG transcript abundances (rho = 0.262 to 0.310, *P* > 0.05, Fig. 1e) and antibiotic concentrations (rho = 0.262 to 0.357, *P* > 0.05, Fig. 1e), the decrease in *tetM*, *tetW*, and *tetO* abundance corresponded with the variations in transcript (*tetM*-*tetW*-*tetO*-*tetS*) level. Consistent with the previous reports where tetracycline resistance genes were undetectable after 28–42 days of composting [14], our study further confirms that composting of raw manure is a potential strategy for not only reducing the abundance but also limiting the expression of tetracycline resistance genes.

### Correlation of ARG-carrying hosts with microbiome profiling during composting

**Fig. 2:**
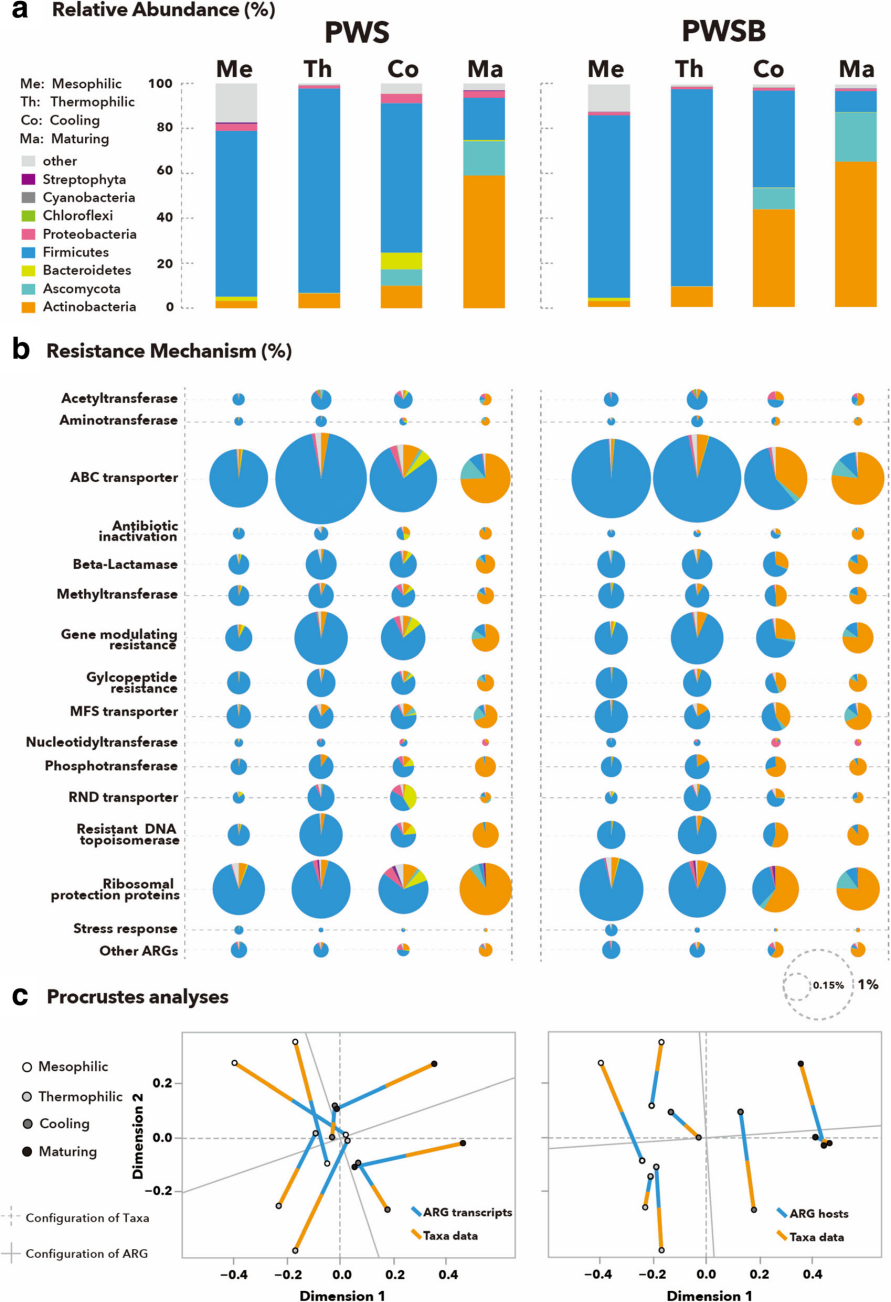
The tightly coordinated transcriptional patterns of active microbiomes and ARG-carrying hosts at the phylum level during the whole composting process in PWS and PWSB. **a** Phylogenetic architectures of active microbiomes predominating the mesophilic, thermophilic, cooling, and maturing phases of composting. **b** Phylogenetic source of expressed resistomes at the resistance mechanism level during the whole composting process; the *bubble size* represents the percentage of the number of sequences assigned to resistance mechanism divided by the total number of sequences. **c** Procrustes analyses show no significant correlation between microbial composition and ARG transcripts (*left*), but indicate significant correlation between the compositions of indigenous microbial communities and ARG-carrying hosts (*right*)

Changes of the resistome are driven by shifts in microbial community composition, a phenomenon recently established for a set of functionally described metagenomic soil samples [16]. The phylogenetic architecture of active microbial consortia and their associated resistome during the composting process were analyzed and compared in the current study. Taxonomic assignment of the cDNA protein-coding gene sequences revealed that Firmicutes, Actinobacteria, Bacteroidetes, and Proteobacteria (all bacteria) as well as Ascomycota (a fungus) were the most transcriptionally active populations in the compost microbiomes (Fig. 2a). The structure of active microbial communities is consistent with previous cultivation-based, metagenomic, and phylogenetic surveys from composted biosolids and lignocellulosic waste [24, 46, 47], suggesting consistency among the most active microbial groups in compost ecosystems. The dynamics of individual taxa revealed a rapid ecological succession. Initially, Firmicutes were predominant in the mesophilic and thermophilic phases and are known to assimilate nitrogenous compounds from manure [48]. Actinobacteria and Ascomycota were dominant in the maturing phase, where these thermophiles can degrade a wide range of recalcitrant organic matter in compost [24, 49]. While Proteobacteria and Bacteroidetes transcripts were present in low abundance, they could potentially complete hemicellulose degradation (via specializing towards its glycosidic linkage hydrolysis) in tandem with Actinobacteria [24]. Consistent with our previous research [12], redundancy analysis showed that the dynamics of the active microbial communities during composting was driven by temperatures (*P* < 0.01), which was likely the key factor controlling the development of diverse niches. The effect of temperature was also evident regarding normal versus biochar-supplemented composting. The biochar-supplemented pile had a relatively greater abundance of Actinobacteria and a lower abundance of Bacteroidetes during the cooling phase. This more closely resembled the maturing phase of composting process. The quicker microbial succession in PWSB further supports enhanced composting kinetics observed with a biochar amendment. This corroborates previous research where intensified humification was observed with biochar amendment [50].

Regarding the phylogenetic source of the resistome, Firmicutes and Actinobacteria were well-represented phyla inferred from the resistance-conferring reads. Importantly, these two phyla contained ARG families encompassing all resistance mechanisms (Fig. 2b). Throughout the composting process, the phylogenetic source of ARG transcripts at the resistance mechanism level was distinct: Firmicutes were the most prevalent phylum harboring ARG families in the mesophilic and thermophilic phases, while the resistome in the matured compost were primarily sourced from Actinobacteria and Ascomycota (Fig. 2b). The Bacteroidetes were the fourth-most abundant phylum containing ARG families in the cooling phase of the PWS pile. They possessed RND transporter, ABC transporter, DNA topoisomerase, and phosphotransferase, as well as gene modulating resistance (Fig. 2b). In addition, among the 16 resistance mechanisms, nucleotidyltransferase was the only type that was not enriched within Firmicutes or Actinobacteria. Proteobacteria, which only accounted for 1.0 to 4.1% of the total active compost microbiomes, were the most prevalent predicted phylum that contained nucleotidyltransferase (ANT2 and ANT3) in the maturing phase. This supports the notion that ANT2 and ANT3 represent the prevalent determinants of aminoglycoside resistance in *Pseudomonas aeruginosa* within the Proteobacteria phylum [51]. Another minor phyla in the active compost microbiomes, Chloroflexi (< 0.1%) which contain aminotransferase (AAC(6*′*)-I), were most prevalent in the thermophilic phase, while Streptophyta (0.1 to 0.6%), which contain tetracycline ribosomal protection proteins, were only detected after the mesophilic phase (Fig. 2b).

Interestingly, a large proportion of the complete resistome displayed the same host succession at the mechanism level and generally matched the dynamics of the active microbiota structure. Bacteroidetes occurred in the cooling phase for both datasets. Such consistency indicates that the core resistome was frequently disseminated among distinct indigenous populations over the course of composting. To further confirm this view, correlations between compost transcriptional resistomes and indigenous microbial community compositions were analyzed on the basis of Bray-Curtis dissimilarity metrics. No highly significant correlations between ARG transcripts and microbial compositions were observed using Procrustes analyses (Fig. 2c: sum of squares *M* ^2^ = 0.9389, *r* = 0.2471, *P* > 0.05, 9999 permutations). By contrast, composition of both indigenous microbiomes and ARG-carrying hosts clustered by composting phases displayed a strong significant correlation (sum of squares *M* ^2^ = 0.4646, *r* = 0.7317, *P* < 0.05, 9999 permutations). Speculatively, the active indigenous microbiome composition and ARG-carrying community composition tend to constitute distinct and complementary axes of variation, with the former being affected strongly by environmental conditions and the latter shaped greatly by community-level processes.

### Roles of microbial phylogeny in determining transcriptional response of resistome

**Fig. 3:**
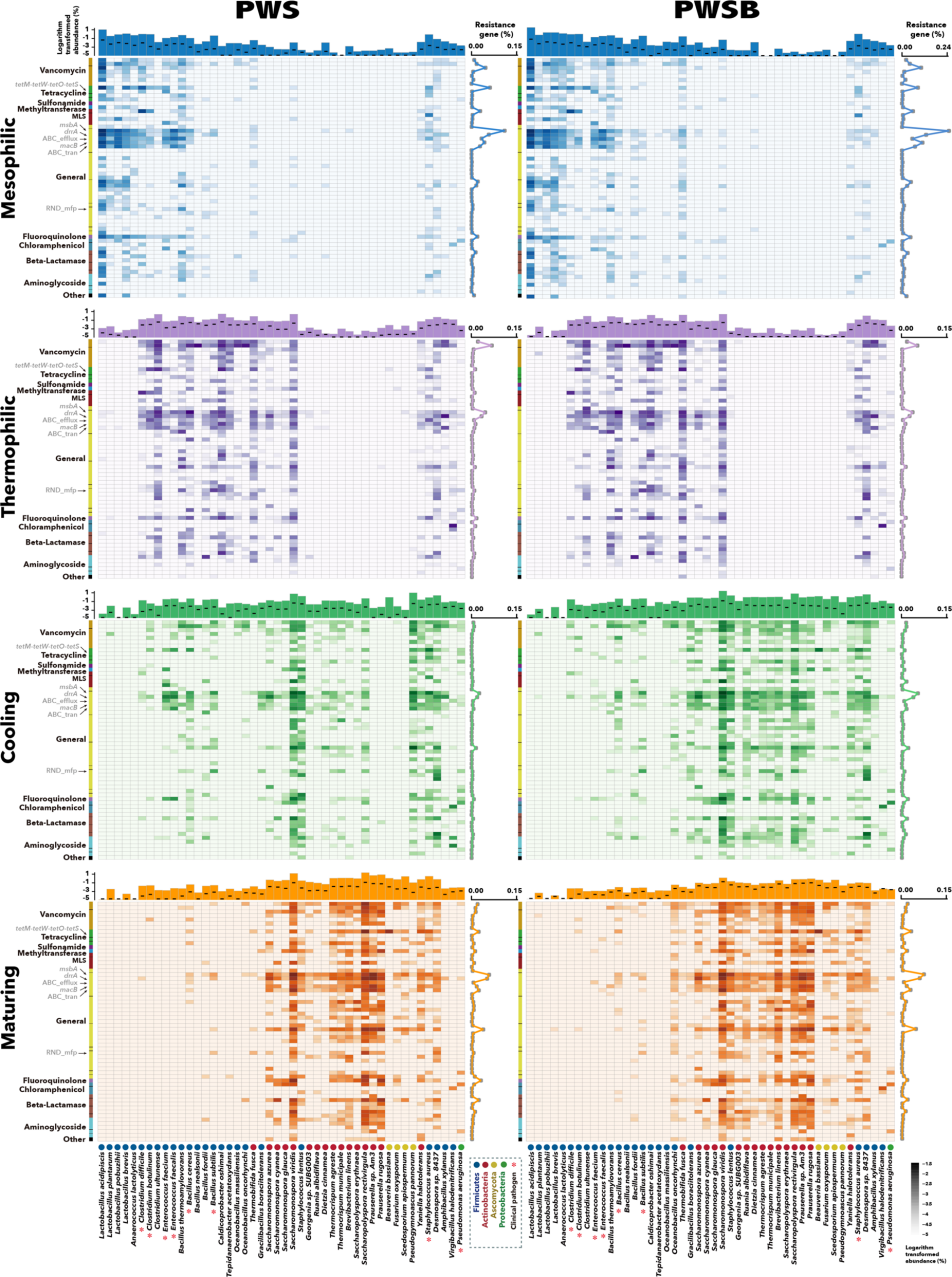
Heatmaps visualize the distribution profiles of ARG-carrying hosts at the species level during composting in PWS and PWSB. Each column and row are labeled with the name of microbial species and dominant resistance gene in the ecosystem. Values plotted are the natural logarithm-transformed proportion of each host carrying one ARG type within the whole microbial community. The diagram above each heatmap shows the natural logarithm-transformed proportion of each species within the whole microbial community, and *black dash* within each bar represents the natural logarithm-transformed proportion of each species carrying ARGs within the whole microbial community. The curve on the right side of each heatmap depicts the aggregated expression levels of ARGs carried by the dominant microbial species listed in the row of the heatmaps. The clinical related pathogen is indicated by an asterisk

A varied response by ARGs to biological treatment has been reported previously [44, 52, 53]. Furthermore, Ma et al. [53] hypothesized that microbial community composition in specific biological treatment systems determined the available ARG hosts, thereby driving the ARG response in sludge digesters. In order to fully examine the roles of indigenous microbiome composition in determining transcriptional response of resistome, a comprehensive taxonomic survey of unique ARG trancript reads was conducted at a higher resolution and the phylogenetic distribution was examined at the species level. Heatmaps revealed that the overall phylogenetic distribution of ARG-carrying communities changed for each of the four composting phases metatranscriptomes (Fig. 3): the relative abundance of resistance-conferring reads assigned to *Lactobacillus* and *Anaerococcus* species declined during the first phase transition, while those assigned to *Clostridium*, *Bacillus*, and *Caldicoprobacter* species increased. After the thermophilic phase, Firmicutes displayed a sharp decrease in the relative abundance, whereas Ascomycota (including *Beauveria*, *Fusarium*, and *Scedosporium* species) and Actinobacteria (*Thermocrispum*, *Brevibacterium*, *Saccharopolyspora*, and *Prauserella* species) became the dominant ARG-carrying hosts until the end of composting. This drastic change in resistome communities also coincided with shifts in microbial metabolic functions. *Lactobacillus* and *Clostridium* species are typical lactic acid-producing and acetate-oxidizing bacteria and are often reported as the dominant consumers of soluble and easily degradable organic matter (such as lipid, protein and starch) present at the onset of composting [54, 55]. By contrast, thermophilic *Saccharomonospora* species predominated during the later phases of composting, presumably as they are able to degrade recalcitrant compounds such as xylan and lignin [56, 57].

As illustrated in the heatmap diagrams (Fig. 3), the abundance of resistance phenotypes and active dominant species shared similar shifts in each phase of the composting process (Spearman rho = 0.836–0.943, *P* value < 0.01). As the ARG-carrying hosts and indigenous species exhibited tightly coordinated transcriptional patterns during composting, it is likely that microbial succession resulting from functional selection played a central role in determining the overall resistome community profiles. Interestingly, these resistome community profiles have a direct effect on the response of resistome to the dynamic composting process. The predominant hosts for sulfonamide and fluoroquinolone resistance genes switched phylogenetically and underwent an initial increase and then a decrease in the total abundance (Fig. 3 and Additional file 7: Figure S5). This shift corresponded well with the weak effect of composting on the expression of sulfonamide and fluoroquinolone resistance genes. By contrast, the hosts for tetracycline resistance genes (*tetM*-*tetW*-*tetO*-*tetS*) were primarily derived from members in the Firmicutes phylum and exhibited a constant decline throughout composting (Additional file 7: Figure S5). *Enterococcus faecium* and *Clostridium botulinum*, which were the most abundant microorganisms carrying *tetM*-*tetW*-*tetO*-*tetS* in Firmicutes, almost disappeared after piles entered the thermophilic phase (Fig. 3). The varieties in resistance phenotypes most likely contributed to the reduced expression of tetracycline resistance genes over composting. Taken together, these findings suggest that microbial phylogeny that drives the resistome community patterns is the key determinant in defining the varied transcriptional response of resistome to a dynamic biological process.

### Advantages of composting in reducing the risk of emerging environmental contaminants

A central concern of elevated antibiotic resistance is the transfer of resistance to pathogens [58]. Repeated application of manure containing antimicrobial agents is capable of causing an enrichment of taxa that include human pathogens [59]. In this study, clinically relevant pathogens carrying ARGs were identified, and the effect of composting on their abundance was evaluated. Of the dominant species, eight belonged to clinically relevant pathogens, namely *Clostridium difficile*, *Clostridium botulinum*, *Enterococcus faecium*, *Enterococcus faecalis*, *Bacillus cereus*, *Bacillus subtilis*, *Staphylococcus aureus*, and *Pseudomonas aeruginosa* (prioritized by the German Robert Koch-Institute; http://www.bode-science-center.com/center/relevant-pathogens-from-a-z.html). While encoding multiple classes of ARGs, they exhibited distinct responses to the composting phases (Fig. 3). For instance, *Staphylococcus aureus* and *Pseudomonas aeruginosa* (carrying tetracycline and MLS and chloramphenicol genes) appeared stable over the entire process. *Clostridium difficile* and *Clostridium botulinum* (tetracycline and MLS resistance genes) were abundant in the mesophilic and thermophilic phases but were negligible in the maturing phase. Similarly, *Enterococcus faecium* and *Enterococcus faecalis* (tetracycline resistance genes and ABC efflux pump) were dominant in the raw manure but disappeared at the end of composting. The abundance of *Bacillus cereus* and *Bacillus subtilis* (ABC and Resistance-nodulation-cell division efflux pump: *msbA*, *ddrA* and RND-MFP, as well as Fluoroquinolone resistance genes) peaked during the active phase of composting but declined rapidly thereafter. Previous research has shown that composts with higher relative proportions of Gram-positive bacteria (such as Actinobacteria) inhibited fecal coliforms such as *Staphylococcus aureus*, *Enterococcus faecalis*, *Escherichia coli*, and *Pseudomonas aeruginosa* [60]. By implication, the indigenous compost microbiome composition may influence pathogen abundance. Thus, a detailed investigation of antimicrobial activity (or microbial competition) within the compost microbiome is worth pursuing in future research. Overall, our observations indicate a substantial decrease in the abundance of many ARG-carrying pathogens over time and suggest that composting is a promising strategy for minimizing the spread of the resistance genes to clinically relevant pathogens. It is also worth noting that based on taxonomic community profiling, a large proportion of viruses (picornavirales and nidovirales) and bacteriophages were killed in the thermophilic phase (Additional file 8: Figure S6), which further supports the beneficial role of thermal inactivation in sanitizing animal manure [61]. It was reported that the efficiency of bacteriophage removal was largely dependent on the temperature of composting, where temperature between 60 to 70 °C completely inactivated bacteriophages from sludge [62]. Taken together, this research highlights composting as a promising technology for reducing the risk of emerging environmental contaminants, while also recycling waste to generate fertilizer, thereby reinforcing the benefits of this biological treatment to livestock manure.

Finally, it is important to note that matured compost is commonly applied as soil conditioner and fertilizer to improve soil quality and plant nutrient. Considering that the persistence of some antibiotics and ARGs was observed after composting, it would be pertinent to determine the fate of the remaining antibiotics and resistant bacteria in the receiving soil ecosystems, as well as their effects on the composition and function of soil microbial communities.

### Potential mechanisms for ARG dissemination during composting and the environmental implication

**Fig. 4:**
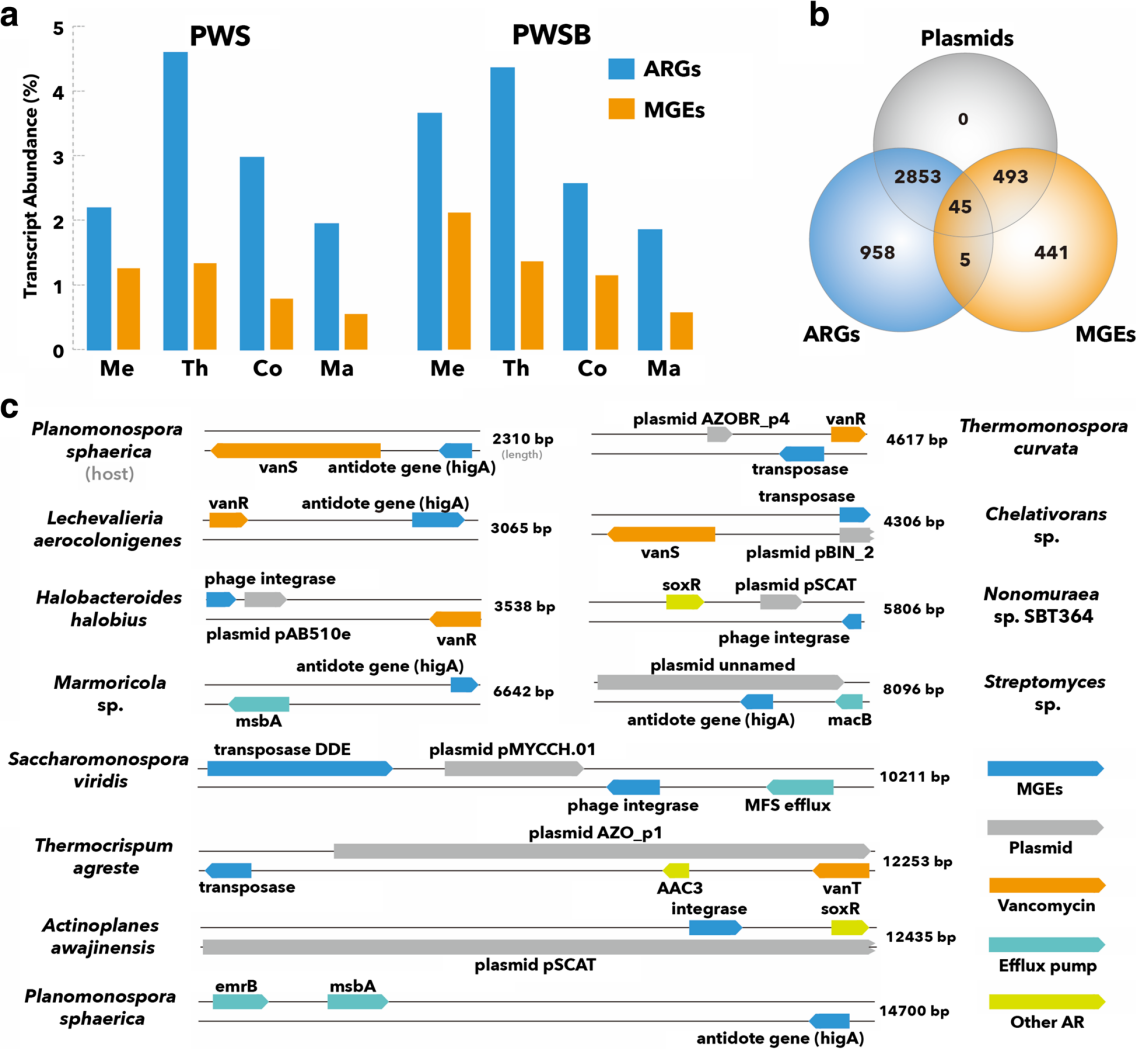
Antibiotic resistance proteins found in genetic contexts of translocative elements in the compost ecosystem. **a** Changes in the expression levels of ARGs and MGEs during the whole composting process in PWS and PWSB. **b** Venn diagram showing the number of metagenomic contigs annotated to antibiotic resistance proteins, translocative elements, and plasmids. **c** Representative alignment of shared ARG-MGE-carrying contigs and their putative hosts (best hits on NCBI)

The exchange of multiple classes of ARGs during composting occurred most frequently among indigenous populations, with frequent host-switching from Firmicutes to Actinobacteria and Ascomycota species. The change in ARG hosts strongly emphasizes the ARGs’ broad host-range compatibility. As many of antibiotic resistance determinants present in microbial genomes were associated with MGEs [8], the occurrence of MGEs was assessed by analyzing genetic sequences in the metatranscriptomic and metagenomic libraries. Annotation of putative MGEs in the metatranscriptomic data indicated a wide range of transcripts involved in genetic transfer (Additional file 9: Table S3). DDE-containing transposase, type IV secretion system, phage integrase, and reverse transcriptase were most frequently detected. Mirroring the varied profile of ARGs transcripts, the abundance of expressed MGEs decreased notably over the course of composting (Fig. 4a). Throughout the process, a significant positive correlation was observed between the number of MGEs and the proportion of resistome (Spearman’s rho = 0.786, *P* < 0.05). This finding suggests a crucial role of translocative elements in antibiotic resistance acquisition and dissemination in the compost ecosystem and is worthy of further study.

To further assess the potential ARG mobility, genetic context of the putative mobile elements and multidrug resistance clusters were assembled, and their location in the metagenomic contigs were determined. Three quarters (75.1%) of all assembled contigs were related to intact or partial plasmids (Fig. 4b), which are key vectors of horizontal gene transfer (HGT) and essential genetic engineering tools [63]. A total of 50 (1.3%) of our unique antibiotic resistance proteins were encoded in more than one translocative elements (as identified using Pfam and TIGRFAM: Fig. 4b). More importantly, a density of resistance genes were flanked by multiple genetic contexts of mobile elements (Fig. 4c). Taking compost-enriched vancomycin resistance genes as an example, they were encoded in three different genetic contexts, including predicted antidote and transposase, as well as phage-related integrase. This is in agreement with literature indicating that the mobile element is rapidly acquired and disseminated in vancomycin-resistant enterococci [64]. Similarly, efflux pumps (*emrB*, *msbA*, and *macB*) were also located in close proximity to genetic contexts of mobile elements within many non-pathogenic Actinobacteria genomes. These pumps expel multiple antibiotics and are recognized as intrinsic mechanisms for multidrug resistance in bacteria [65]. The genetic association between particular ARGs and MGEs, along with the corresponding contig host tracking, implies that the prevalence of MGEs promoted the acquisition and dissemination of resistance traits via HGT among a diverse range of hosts. This may provide an explanation for the fact that the “new” developed dominant populations could be well adapted to environments with an antibiotic stress. Consequently, tracking ARGs and the associated MGEs, rather than microorganisms, appears to be the more appropriate method to identify major routes of resistance dissemination and to assess future clinical risks. Given that ARGs are prevalent for extended time periods in an environment, it is increasingly clear that implementing programs and policies that limit the use of antibiotics, and the discovery of effective antibiotic alternatives, are urgently needed to prevent the development and dissemination of ARGs, while protecting the environment and human health.

## Conclusions

The present study is the first research defining the transcriptional response of resistome to a dynamic biological process through integrated hosts and microbiome profiling. Our data confirm a varied transcriptional response of ARGs to composting and demonstrate that microbial phylogeny is the key determinant in defining the varied transcriptional response of resistome to a dynamic biological process. Additionally, the prevalence and diversity of mobile genetic elements further support the pivotal role of translocative elements in disseminating ARGs among a diverse range of hosts in the compost ecosystem. Composting noticeably reduced the aggregated expression level of the manure resistome and lowered the risk of emerging environmental contaminants, including tetracyclines, tetracycline resistance genes, and clinically relevant pathogens carrying ARGs, as well as RNA viruses and bacteriophages.

## Additional files

Additional file 1: Figure S1. Changes in temperature of composting material during the manure composting process. (PDF 259 kb)

Additional file 2: Table S1. Summary statistics for compost metatranscriptome datasets. (DOCX 20.4 kb)

Additional file 3: Table S2. Primers and PCR conditions in this study. (DOCX 23.3 kb)

Additional file 4: Figure S2. Changes in expression levels of the top 80 ARG types during the whole composting process. Circle represents the average value of relative abundance of each expressed ARG types from PWS and PWSB piles. (PDF 0.99 kb)

Additional file 5: Figure S3. The non-metric multidimensional scaling (NMDS) ordination at the resistance class, mechanism and ARGs levels using Euclidean distances. (PDF 224 kb)

Additional file 6: Figure S4. The varied transcriptional responses of ARGs to composting treatment. The highlighted bar represents each of composting phase where the ARGs were highest expressed. (PDF 769 kb)

Additional file 7: Figure S5. Changes in the abundance of hosts for tetracycline, sulfonamide, and fluoroquinolone resistance genes during the whole composting process. (PDF 23.8 kb)

Additional file 8: Figure S6. Changes in the relative abundance of virus (A) and bacteriophage (B) over the whole composting process. The relative abundance of the virus was calculated as the percentage of the number of sequences assigned to this taxon divided by the total number of sequences assigned to all the taxa in the community. (PDF 740 kb)

Additional file 9: Table S3. The relative abundance of mobile genetic elements (MGEs) during the whole composting process in PWS and PWSB. (DOCX 43.1 kb)

## Abbreviations

ARG: Antibiotic resistance gene
CFC: Ciprofloxacin
CTC: Chlortetracycline
DOC: Doxycycline
EFC: Enrofloxacin
FQ: Fluoroquinolone
HGT: Horizontal gene transfer
MGE: Mobile genetic element
NFC: Norfloxacin
OFC: Ofloxacin
OTC: Oxytetracycline
SA: Sulfonamide
SCZ: Sulfaclozine
SDZ: Sulfadiazine
SMX: Sulfamethoxazole
SMZ: Sulfamerazine
TC: Tetracycline
