# Supplementary figures and images for: Microbial phylogeny determines transcriptional response of resistome to dynamic composting processes
Source: Microbiome. 2017 Aug 16;5:103. doi: 10.1186/s40168-017-0324-0 (PMC5559795; doi:10.1186/s40168-017-0324-0)

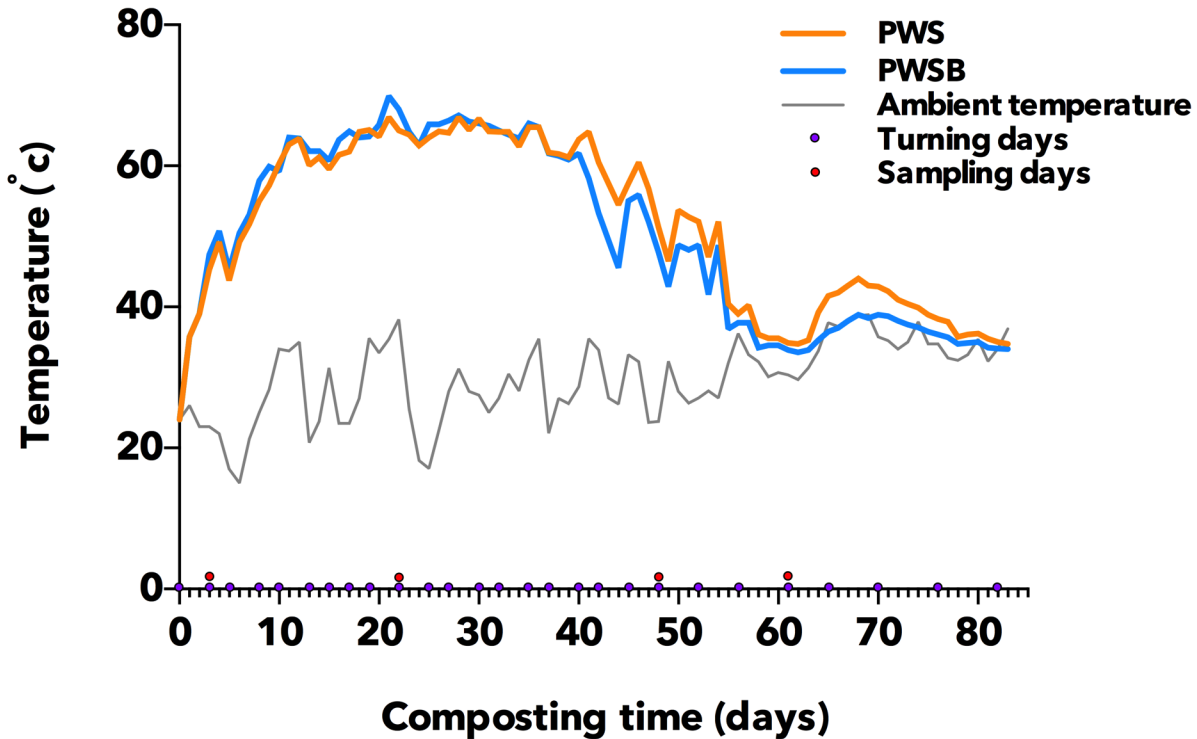

Supplement: Supplementary file 1 — Changes in temperature of composting material during the manure composting process. (PDF 259 kb) [file 40168_2017_324_MOESM1_ESM.pdf]

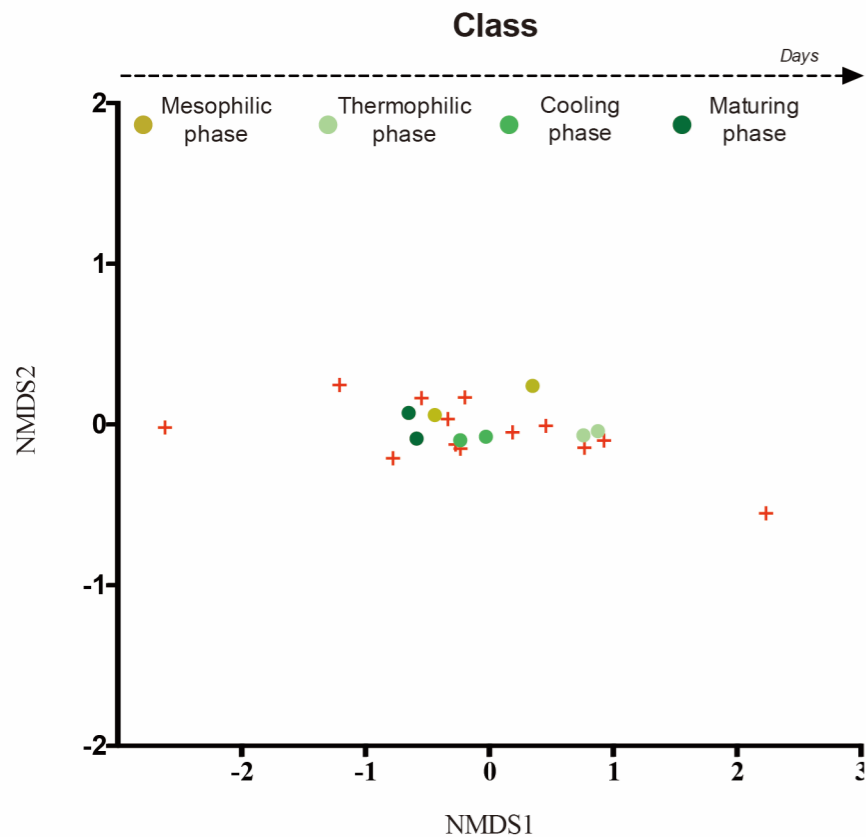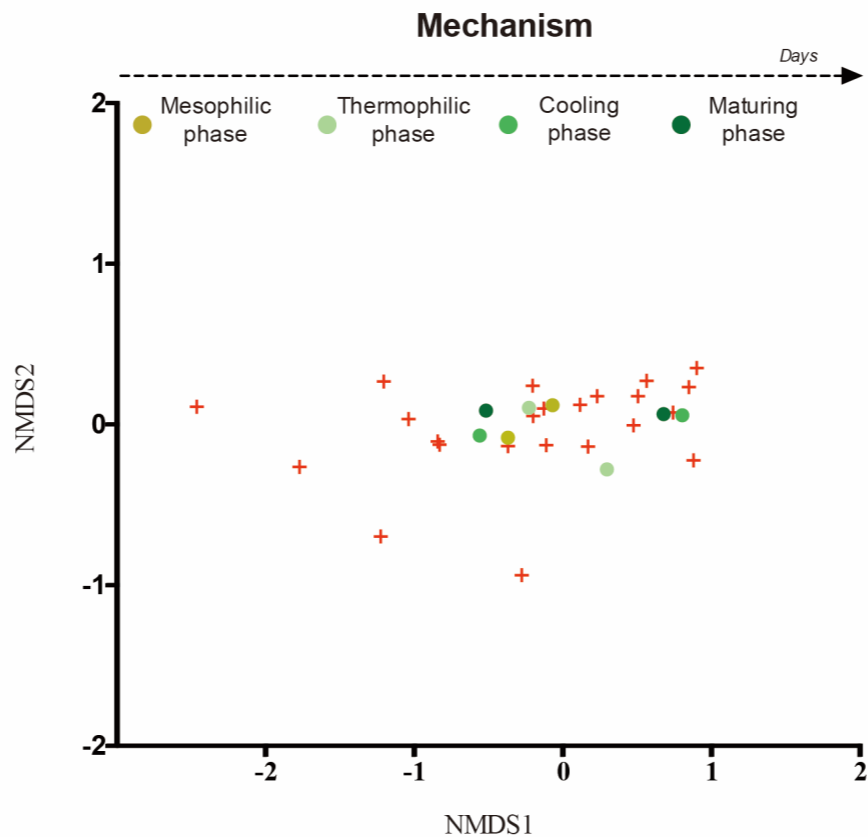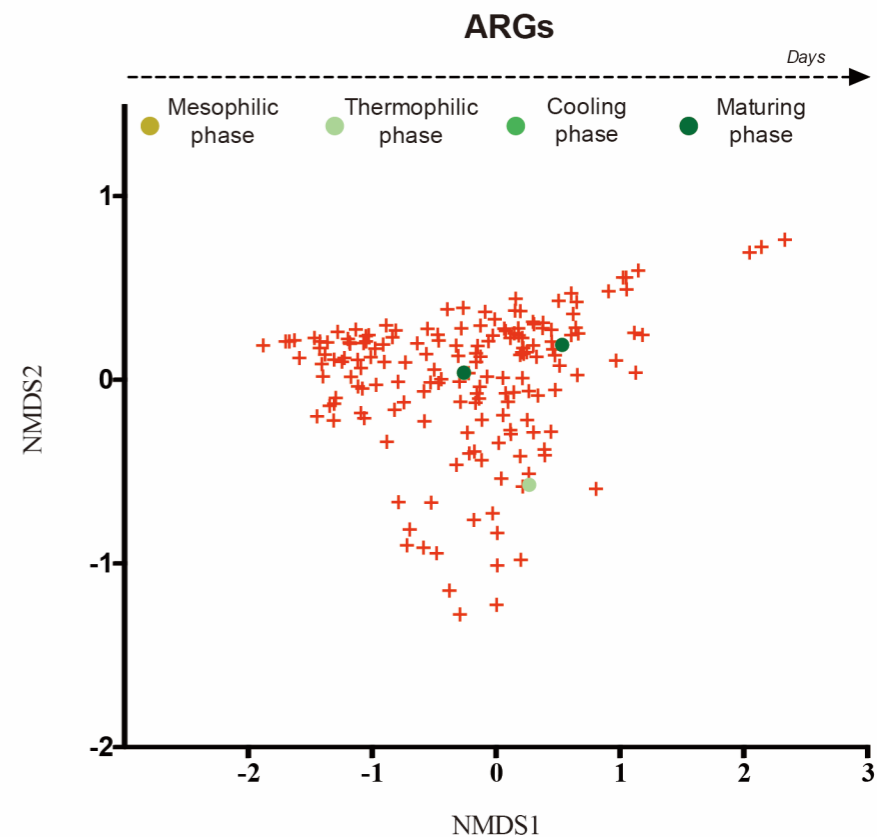

Supplement: Supplementary file 5 — The non-metric multidimensional scaling (NMDS) ordination at the resistance class, mechanism and ARGs levels using Euclidean distances. (PDF 224 kb) [file 40168_2017_324_MOESM5_ESM.pdf]

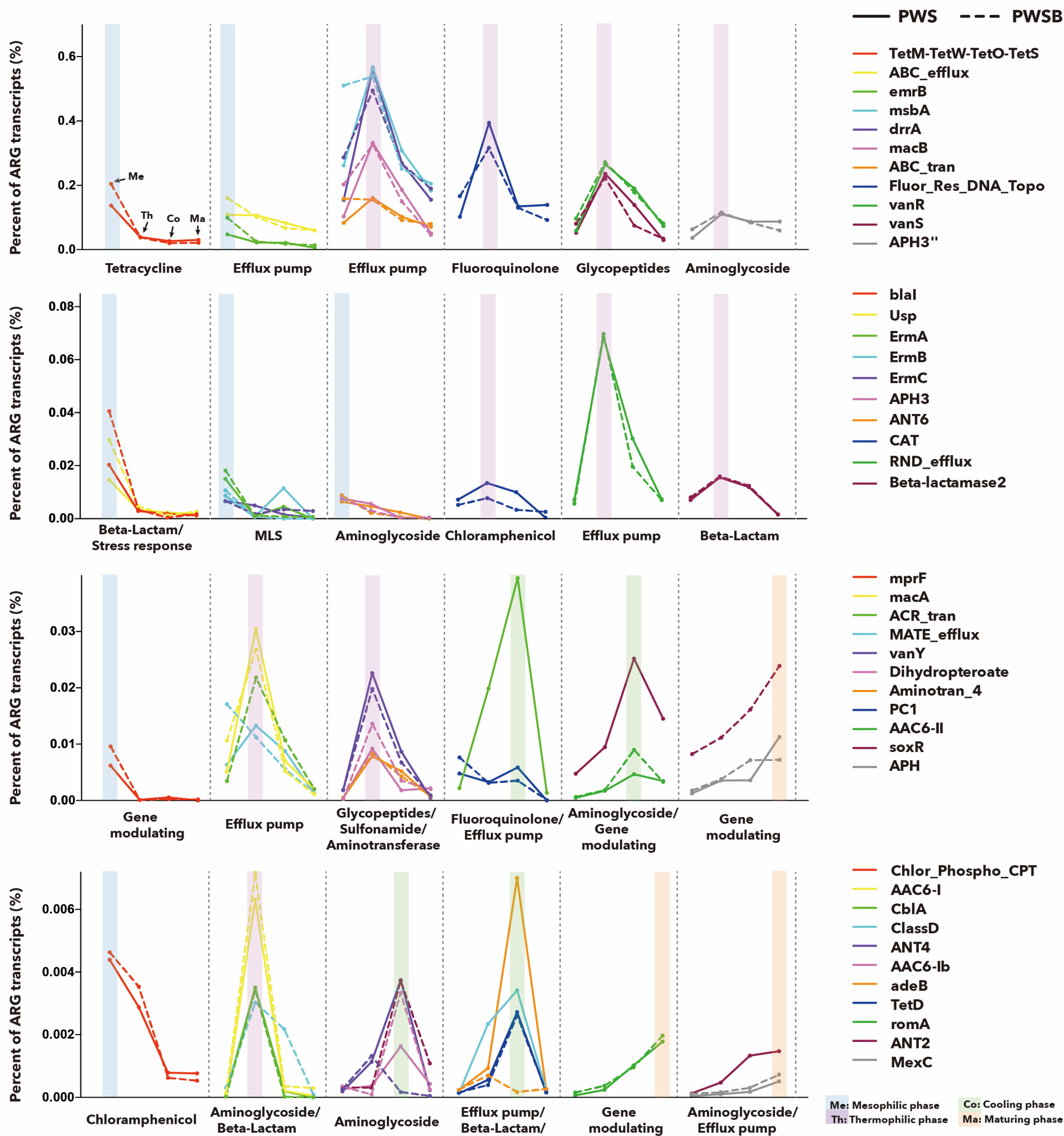

Supplement: Supplementary file 6 — The varied transcriptional responses of ARGs to composting treatment. The highlighted bar represents each of composting phase where the ARGs were highest expressed. (PDF 769 kb) [file 40168_2017_324_MOESM6_ESM.pdf]

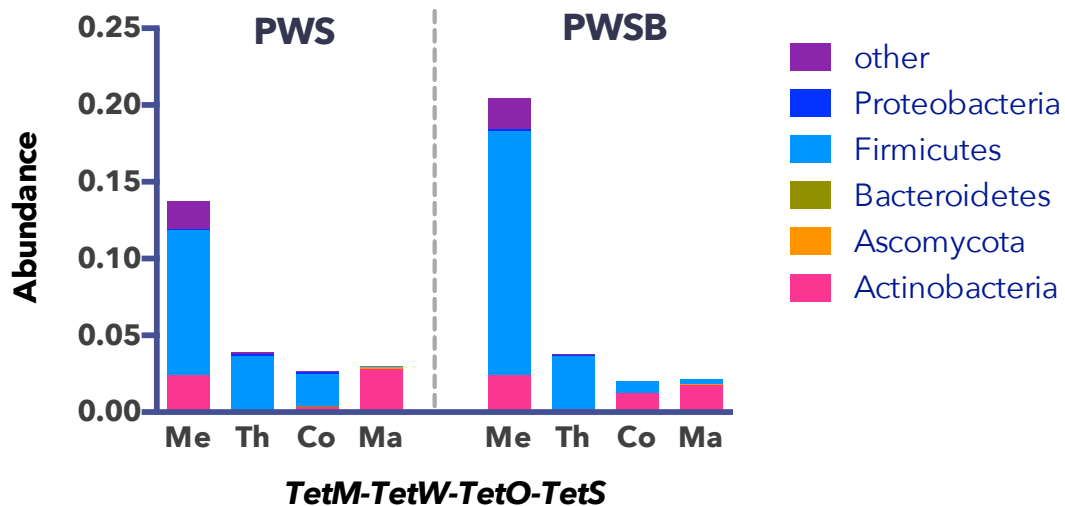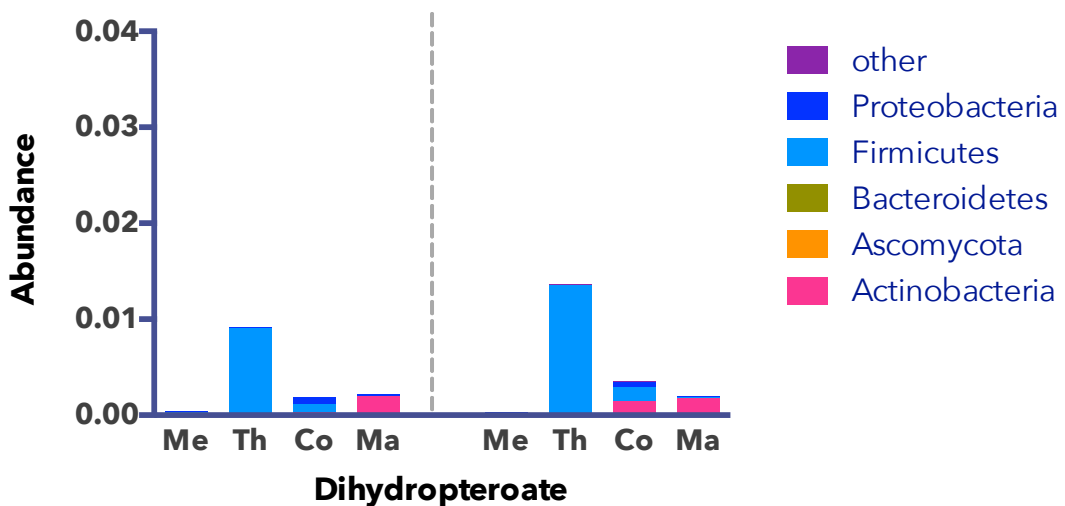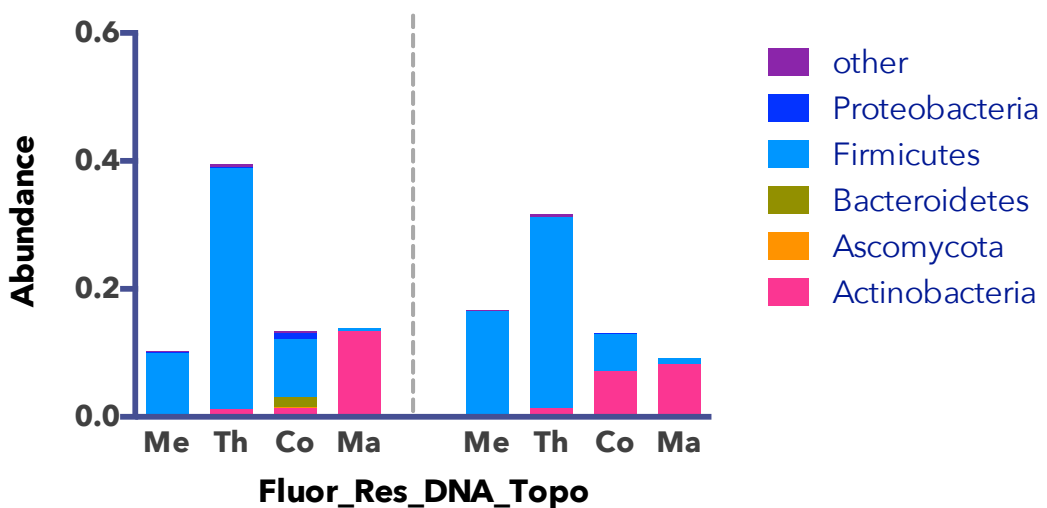

Supplement: Supplementary file 7 — Changes in the abundance of hosts for tetracycline, sulfonamide, and fluoroquinolone resistance genes during the whole composting process. (PDF 23.8 kb) [file 40168_2017_324_MOESM7_ESM.pdf]

### (A) Virus

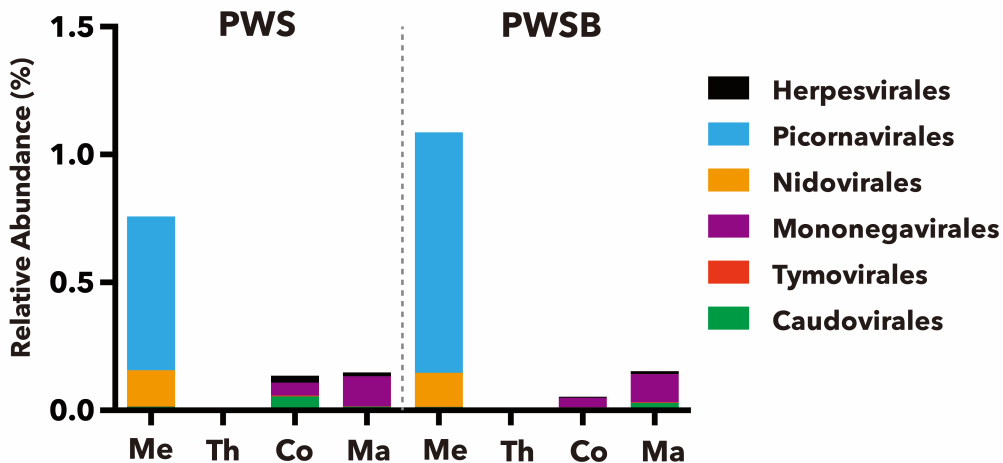

## (B) Bacteriophage

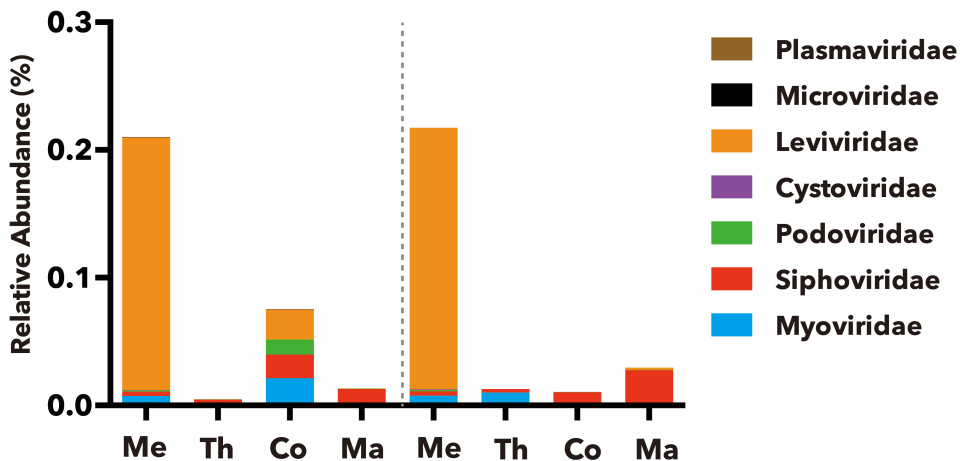

Supplement: Supplementary file 8 — Changes in the relative abundance of virus (A) and bacteriophage (B) over the whole composting process. The relative abundance of the virus was calculated as the percentage of the number of sequences assigned to this taxon divided by the total number of sequences assigned to all the taxa in the community. (PDF 740 kb) [file 40168_2017_324_MOESM8_ESM.pdf]
