# Supplementary material for: Microbial phylogeny determines transcriptional response of resistome to dynamic composting processes
Source: Microbiome. 2017 Aug 16;5:103. doi: 10.1186/s40168-017-0324-0 (PMC5559795; doi:10.1186/s40168-017-0324-0)
Supplement: Supplementary file 2 — Summary statistics for compost metatranscriptome datasets. (DOCX 20.4 kb) [file 40168_2017_324_MOESM2_ESM.docx]

**Table S1. Summary statistics for compost metatranscriptome datasets**

|  | **PWS**  **Me** | **PWSB**  **Me** | **PWS**  **Th** | **PWSB**  **Th** | **PWS**  **Co** | **PWSB**  **Co** | **PWS**  **Ma** | **PWSB**  **Ma** |
| --- | --- | --- | --- | --- | --- | --- | --- | --- |
| **Total reads** | 3,697,472 | 3,310,358 | 4,756,100 | 4,156,013 | 3,840,813 | 3,790,038 | 3,864,157 | 5,504,815 |
| **Average length** | 315 bp | 363 bp | 358 bp | 377 bp | 356 bp | 375 bp | 367 bp | 337 bp |
| **Mean GC** | 44 ± 8 % | 44 ± 7 % | 43 ± 9 % | 46 ± 10 % | 46 ± 10 % | 54 ± 13 % | 58 ± 12 % | 54 ± 15 % |
| **Predicted feature** | 41.7% | 48.6% | 59.3% | 64.8% | 52.3% | 48.3% | 33.4% | 42.3% |
| **Annotated protein in predicted feature** | 41.9% | 53.5% | 91.4% | 92.3% | 78.8% | 73.8% | 36.5% | 53.7% |
| **Ribosomal RNA in predicted feature** | 58.1% | 46.5% | 8.6% | 7.7% | 21.2% | 26.2% | 63.5% | 46.3% |

Note: “Me”, “Th”, “Co”, and “Ma” respresent the mesophilic, thermophilic, cooling and maturing phase, respectively.
