# Supplementary material for: Microbial phylogeny determines transcriptional response of resistome to dynamic composting processes
Source: Microbiome. 2017 Aug 16;5:103. doi: 10.1186/s40168-017-0324-0 (PMC5559795; doi:10.1186/s40168-017-0324-0)
Supplement: Supplementary file 3 — Primers and PCR conditions in this study. (DOCX 23.3 kb) [file 40168_2017_324_MOESM3_ESM.docx]

**Table S2. Primers and PCR conditions in this study**

| Target  genes | Primer | Sequences | Annealing  Temp. (℃) | References |
| --- | --- | --- | --- | --- |
| *tetM* | *tetM*-F | ACAGAAAGCTTATTATATAAC | 55 | [1] |
|  | *tetM*-R | TGGCGTGTCTATGATGTTCAC |  |  |
| *tetO* | *tetO*-F | GATGGCATACAGGCACAGACC | 57 | [2] |
|  | *tetO*-R | GCCCAACCTTTTGCTTCACTA |  |  |
| *tetQ* | *tetQ*-F | AGAATCTGCTGTTTGCCAGTG | 62 | [1] |
|  | *tetQ*-R | CGGAGTGTCAATGATATTGCA |  |  |
| *tetW* | *tetW*-F | GAGAGCCTGCTATATGCCAGC | 60 | [1] |
|  | *tetW*-R | GGGCGTATCCACAATGTTAAC |  |  |
| *sulI* | *sulI*-F | CACCGGAAACATCGCTGCA | 55 | [2] |
|  | *sulI*-R | AAGTTCCGCCGCAAGGCT |  |  |
| *sulII* | *sulII*-F | CTCCGATGGAGGCCGGTAT | 60 | [2] |
|  | *sulII*-R | GGGAATGCCATCTGCCTTGA |  |  |
| *intI1* | *intI1*-F | CCTCCCGCACGATGATC | 63 | [3] |
|  | *intI1*-R | TCCACGCATCGTCAGGC |  |  |

**Reference**

[1] Aminov R, Garrigues-Jeanjean N, Mackie R. Molecular ecology of tetracycline resistance: development and validation of primers for detection of tetracycline resistance genes encoding ribosomal protection proteins. Appl Environ Microbiol. 2001;67(1): 22-32.

[2] Luo Y, Mao DQ, Rysz M, Zhou Q, Zhang H, Xu L et al. Trends in antibiotic resistance genes occurrence in the Haihe river, China. Environ Sci Technol. 2010;44(19): 7220-7225.

[3] Goldstein C, Lee MD, Sanchez S, Hudson C, Phillips B, Register B et al. Incidence of class 1 and 2 integrases in clinical and commensal bacteria from livestock, companion animals, and exotics. Antimicrob Agents Chemother. 2001;45(3): 723-726.
