# Supplementary material for: Microbial phylogeny determines transcriptional response of resistome to dynamic composting processes
Source: Microbiome. 2017 Aug 16;5:103. doi: 10.1186/s40168-017-0324-0 (PMC5559795; doi:10.1186/s40168-017-0324-0)
Supplement: Supplementary file 9 — The relative abundance of mobile genetic elements (MGEs) during the whole composting process in PWS and PWSB. (DOCX 43.1 kb) [file 40168_2017_324_MOESM9_ESM.docx]

**Table S3. The relative abundance of mobile genetic elements (MGEs) during the whole composting process in PWS and PWSB**

| Source | Profile HMM | Annotation | Relative abundance (%) | | | | | | | |
| --- | --- | --- | --- | --- | --- | --- | --- | --- | --- | --- |
|  |  |  | PWS  Me | PWSB  Me | PWS  Th | PWSB  Th | PWS  Co | PWSB  Co | PWS  Ma | PWSB  Ma |
| Pfam | PF00078 | Reverse transcriptase (RNA-dependent DNA polymerase) | 0.070 | 0.139 | 0.025 | 0.024 | 0.018 | 0.208 | 0.015 | 0.030 |
| Pfam | PF00589 | Phage integrase family | 0.058 | 0.095 | 0.086 | 0.083 | 0.054 | 0.046 | 0.029 | 0.035 |
| Pfam | PF00665 | Integrase core domain | 0.056 | 0.103 | 0.082 | 0.079 | 0.041 | 0.050 | 0.023 | 0.025 |
| Pfam | PF00872 | Transposase. Mutator family | 0.037 | 0.073 | 0.079 | 0.086 | 0.031 | 0.040 | 0.009 | 0.012 |
| Pfam | PF01076 | Plasmid recombination enzyme | 0.030 | 0.027 | 0.017 | 0.013 | 0.006 | 0.004 | 0.002 | 0.005 |
| Pfam | PF01385 | Probable transposase | 0.015 | 0.013 | 0.071 | 0.068 | 0.013 | 0.017 | 0.005 | 0.003 |
| Pfam | PF01526 | Tn3 transposase DDE domain | 0.001 | 0.002 | 0.002 | 0.003 | 0.002 | 0.008 | 0.005 | 0.006 |
| Pfam | PF01527 | Transposase | 0.019 | 0.030 | 0.057 | 0.057 | 0.032 | 0.030 | 0.017 | 0.018 |
| Pfam | PF01548 | Transposase | 0.019 | 0.037 | 0.050 | 0.048 | 0.026 | 0.042 | 0.007 | 0.011 |
| Pfam | PF01609 | Transposase DDE domain | 0.025 | 0.034 | 0.066 | 0.079 | 0.030 | 0.051 | 0.022 | 0.015 |
| Pfam | PF01610 | Transposase | 0.053 | 0.115 | 0.039 | 0.037 | 0.009 | 0.011 | 0.003 | 0.008 |
| Pfam | PF01797 | Transposase IS200 like | 0.004 | 0.006 | 0.009 | 0.008 | 0.004 | 0.006 | 0.001 | 0.001 |
| Pfam | PF02371 | Transposase IS116/IS110/IS902 family | 0.014 | 0.031 | 0.037 | 0.033 | 0.018 | 0.028 | 0.007 | 0.012 |
| Pfam | PF02534 | Type IV secretory system Conjugative DNA transfer | 0.018 | 0.032 | 0.009 | 0.011 | 0.008 | 0.009 | 0.006 | 0.008 |
| Pfam | PF02604 | Antitoxin Phd_YefM. type II toxin-antitoxin system | 0.007 | 0.007 | 0.022 | 0.024 | 0.005 | 0.007 | 0.006 | 0.007 |
| Pfam | PF02661 | Fic/DOC family | 0.009 | 0.015 | 0.003 | 0.003 | 0.006 | 0.005 | 0.002 | 0.004 |
| Pfam | PF02899 | Phage integrase. N-terminal SAM-like domain | 0.012 | 0.018 | 0.024 | 0.024 | 0.015 | 0.014 | 0.010 | 0.010 |
| Pfam | PF02993 | Minor capsid protein VI | 0.003 | 0.007 | 0.003 | 0.002 | 0.003 | 0.001 | 0.001 | 0.001 |
| Pfam | PF03050 | Transposase IS66 family | 0.027 | 0.052 | 0.008 | 0.009 | 0.012 | 0.010 | 0.005 | 0.005 |
| Pfam | PF03354 | Phage Terminase | 0.003 | 0.004 | 0.004 | 0.003 | 0.004 | 0.006 | 0.001 | 0.002 |
| Pfam | PF03389 | MobA/MobL family | 0.009 | 0.011 | 0.005 | 0.005 | 0.001 | 0.000 | 0.000 | 0.000 |
| Pfam | PF03400 | IS1 transposase | 0.001 | 0.001 | 0.003 | 0.003 | 0.002 | 0.000 | 0.000 | 0.001 |
| Pfam | PF03432 | Relaxase/Mobilisation nuclease domain | 0.039 | 0.036 | 0.001 | 0.002 | 0.001 | 0.002 | 0.002 | 0.002 |
| Pfam | PF04221 | RelB antitoxin | 0.040 | 0.033 | 0.003 | 0.004 | 0.003 | 0.002 | 0.002 | 0.005 |
| Pfam | PF04693 | Archaeal putative transposase ISC1217 | 0.002 | 0.001 | 0.001 | 0.001 | 0.001 | 0.001 | 0.000 | 0.000 |
| Pfam | PF04986 | Putative transposase | 0.002 | 0.001 | 0.001 | 0.000 | 0.003 | 0.002 | 0.000 | 0.000 |
| Pfam | PF05015 | Plasmid maintenance system killer protein | 0.005 | 0.004 | 0.001 | 0.001 | 0.002 | 0.001 | 0.001 | 0.006 |
| Pfam | PF05016 | Plasmid stabilisation system protein | 0.017 | 0.015 | 0.003 | 0.001 | 0.005 | 0.002 | 0.002 | 0.004 |
| Pfam | PF05598 | Transposase domain (DUF772) | 0.011 | 0.011 | 0.044 | 0.043 | 0.011 | 0.014 | 0.002 | 0.002 |
| Pfam | PF05709 | Phage tail protein | 0.001 | 0.002 | 0.005 | 0.003 | 0.005 | 0.008 | 0.001 | 0.001 |
| Pfam | PF05713 | Bacterial mobilisation protein (MobC) | 0.024 | 0.017 | 0.002 | 0.001 | 0.001 | 0.002 | 0.001 | 0.001 |
| Pfam | PF05717 | IS66 Orf2 like protein | 0.008 | 0.020 | 0.012 | 0.009 | 0.003 | 0.004 | 0.002 | 0.001 |
| Pfam | PF06605 | Prophage endopeptidase tail | 0.001 | 0.003 | 0.007 | 0.006 | 0.003 | 0.005 | 0.001 | 0.001 |
| Pfam | PF07022 | Bacteriophage CI repressor helix-turn-helix domain | 0.014 | 0.015 | 0.014 | 0.014 | 0.009 | 0.011 | 0.004 | 0.004 |
| Pfam | PF07275 | Antirestriction protein (ArdA) | 0.001 | 0.001 | 0.001 | 0.001 | 0.001 | 0.001 | 0.000 | 0.000 |
| Pfam | PF07508 | Recombinase | 0.017 | 0.026 | 0.005 | 0.004 | 0.004 | 0.006 | 0.005 | 0.006 |
| Pfam | PF09720 | Putative addiction module component | 0.001 | 0.001 | 0.001 | 0.001 | 0.002 | 0.001 | 0.003 | 0.003 |
| Pfam | PF10412 | Type IV secretion-system coupling protein DNA-binding domain | 0.126 | 0.216 | 0.133 | 0.134 | 0.093 | 0.088 | 0.065 | 0.082 |
| Pfam | PF10551 | MULE transposase domain | 0.000 | 0.001 | 0.002 | 0.002 | 0.001 | 0.003 | 0.004 | 0.001 |
| Pfam | PF12642 | Conjugative transposon protein TcpC | 0.005 | 0.012 | 0.004 | 0.005 | 0.002 | 0.004 | 0.000 | 0.001 |
| Pfam | PF12696 | TraM recognition site of TraD and TraG | 0.003 | 0.004 | 0.001 | 0.002 | 0.003 | 0.005 | 0.013 | 0.015 |
| Pfam | PF12784 | PD-(D/E)XK nuclease family transposase | 0.005 | 0.004 | 0.003 | 0.003 | 0.002 | 0.001 | 0.000 | 0.000 |
| Pfam | PF12835 | Integrase | 0.000 | 0.003 | 0.001 | 0.001 | 0.000 | 0.000 | 0.000 | 0.000 |
| Pfam | PF13011 | leucine-zipper of insertion element IS481 | 0.008 | 0.012 | 0.004 | 0.004 | 0.003 | 0.005 | 0.005 | 0.008 |
| Pfam | PF13333 | Integrase core domain | 0.006 | 0.008 | 0.014 | 0.012 | 0.007 | 0.004 | 0.002 | 0.003 |
| Pfam | PF13340 | Putative transposase of IS4/5 family (DUF4096) | 0.004 | 0.012 | 0.001 | 0.002 | 0.004 | 0.007 | 0.011 | 0.014 |
| Pfam | PF13495 | Phage integrase. N-terminal SAM-like domain | 0.005 | 0.006 | 0.006 | 0.006 | 0.006 | 0.010 | 0.006 | 0.013 |
| Pfam | PF13542 | Helix-turn-helix domain of transposase family ISL3 | 0.066 | 0.123 | 0.099 | 0.127 | 0.083 | 0.117 | 0.076 | 0.065 |
| Pfam | PF13546 | DDE superfamily endonuclease | 0.008 | 0.014 | 0.007 | 0.016 | 0.004 | 0.007 | 0.008 | 0.009 |
| Pfam | PF13586 | Transposase DDE domain | 0.003 | 0.007 | 0.008 | 0.010 | 0.004 | 0.008 | 0.007 | 0.009 |
| Pfam | PF13610 | DDE domain | 0.028 | 0.053 | 0.030 | 0.024 | 0.015 | 0.036 | 0.054 | 0.014 |
| Pfam | PF13612 | Transposase DDE domain | 0.005 | 0.008 | 0.002 | 0.002 | 0.001 | 0.000 | 0.000 | 0.000 |
| Pfam | PF13683 | Integrase core domain | 0.011 | 0.022 | 0.022 | 0.023 | 0.010 | 0.012 | 0.009 | 0.011 |
| Pfam | PF13701 | Transposase DDE domain group 1 | 0.144 | 0.389 | 0.034 | 0.040 | 0.049 | 0.083 | 0.016 | 0.010 |
| Pfam | PF13737 | Transposase DDE domain | 0.000 | 0.000 | 0.002 | 0.001 | 0.000 | 0.000 | 0.000 | 0.000 |
| Pfam | PF13751 | Transposase DDE domain | 0.008 | 0.010 | 0.012 | 0.010 | 0.006 | 0.005 | 0.001 | 0.001 |
| Pfam | PF13808 | DDE_Tnp_1-associated | 0.001 | 0.001 | 0.003 | 0.003 | 0.002 | 0.002 | 0.002 | 0.001 |
| Pfam | PF14198 | Transposon-encoded protein TnpV | 0.003 | 0.004 | 0.002 | 0.002 | 0.001 | 0.001 | 0.001 | 0.001 |
| Pfam | PF14202 | Transposon-encoded protein TnpW | 0.001 | 0.002 | 0.001 | 0.001 | 0.000 | 0.000 | 0.001 | 0.001 |
| Pfam | PF14319 | Transposase zinc-binding domain | 0.007 | 0.010 | 0.009 | 0.008 | 0.010 | 0.005 | 0.003 | 0.004 |
| TIGRFam | TIGR02607 | antidote_HigA: addiction module antidote protein. HigA family | 0.029 | 0.036 | 0.045 | 0.042 | 0.041 | 0.050 | 0.047 | 0.035 |
| TIGRFam | TIGR01665 | put_anti_recept: phage minor structural protein. N-terminal region | 0.002 | 0.002 | 0.005 | 0.004 | 0.004 | 0.004 | 0.001 | 0.001 |
| TIGRFam | TIGR02384 | RelB_DinJ: addiction module antitoxin. RelB/DinJ family | 0.042 | 0.033 | 0.003 | 0.003 | 0.003 | 0.002 | 0.002 | 0.002 |
| TIGRFam | TIGR02249 | integrase_gron: integron integrase | 0.039 | 0.061 | 0.081 | 0.080 | 0.038 | 0.034 | 0.018 | 0.018 |
| TIGRFam | TIGR02385 | RelE_StbE: addiction module toxin. RelE/StbE family | 0.025 | 0.026 | 0.005 | 0.004 | 0.004 | 0.001 | 0.000 | 0.001 |

Note: “Me”, “Th”, “Co”, and “Ma” respresent the mesophilic, thermophilic, cooling and maturing phase, respectively.
