# Supplementary material for: Microbial phylogeny determines transcriptional response of resistome to dynamic composting processes
Source: Microbiome. 2017 Aug 16;5:103. doi: 10.1186/s40168-017-0324-0 (PMC5559795; doi:10.1186/s40168-017-0324-0)
Supplement: Supplementary file 4 — Changes in expression levels of the top 80 ARG types during the whole composting process. Circle represents the average value of relative abundance of each expressed ARG types from PWS and PWSB piles. (PDF 0.99 kb) [file 40168_2017_324_MOESM4_ESM.pdf]

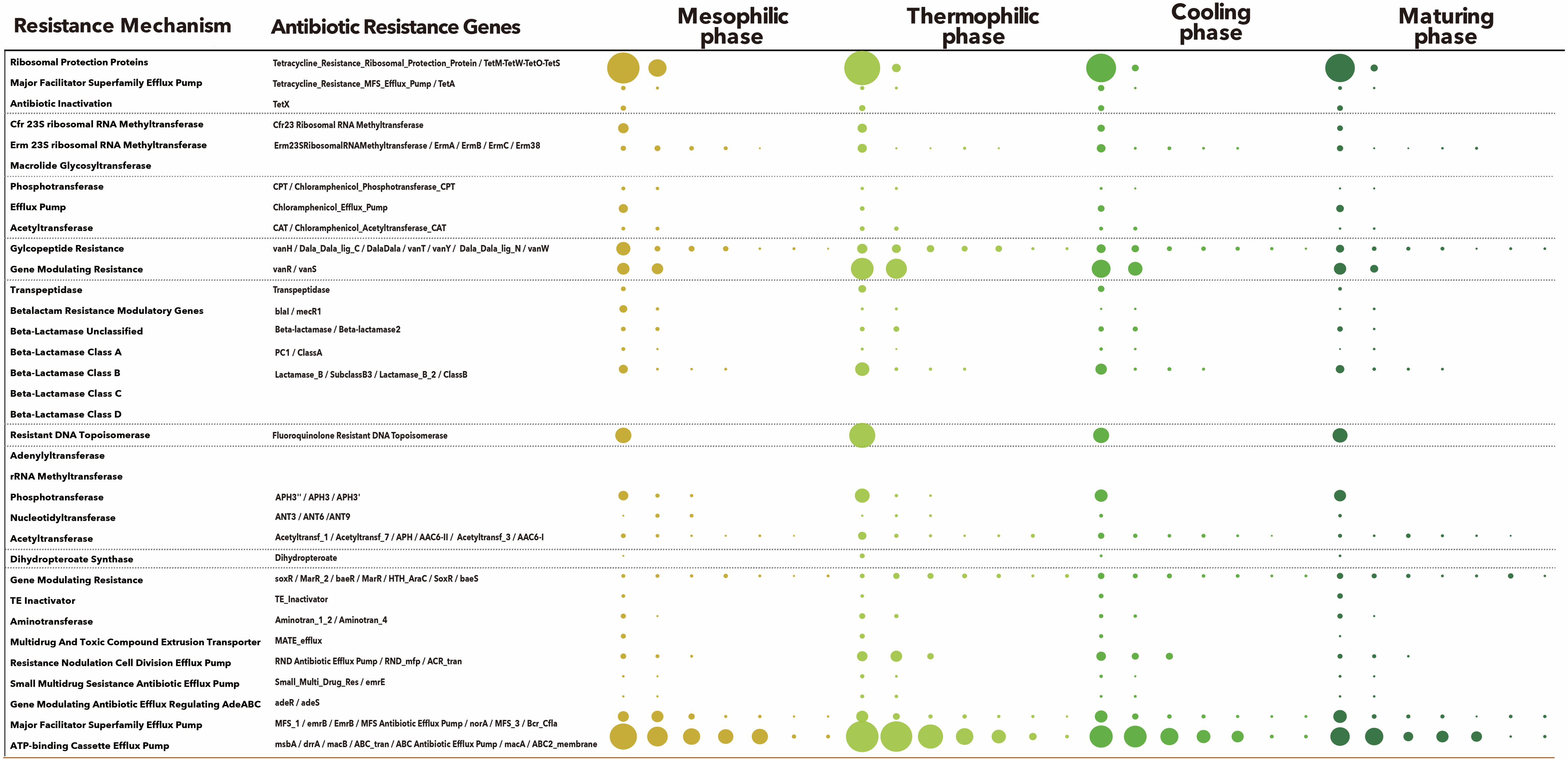

Resistance Mechanism
Antibiotic Resistance Genes
Mesophilic phase
Thermophilic phase
Cooling phase
Maturing phase
Ribosomal Protection Proteins
Tetracycline_Resistance_Ribosomal_Protection_Protein / TetM-TetW-TetO-TetS
Major Facilitator Superfamily Efflux Pump
Tetracycline_Resistance_MFS_Efflux_Pump / TetA
Antibiotic Inactivation
TetX
Cfr 23S ribosomal RNA Methyltransferase
Cfr23 Ribosomal RNA Methyltransferase
Erm 23S ribosomal RNA Methyltransferase
Erm23SRibosomalRNAMethyltransferase / ErmA / ErmB / ErmC / Erm38
Macrolide Glycosyltransferase
Phosphotransferase
CPT / Chloramphenicol_Phosphotransferase_CPT
Efflux Pump
Chloramphenicol_Efflux_Pump
Acetyltransferase
CAT / Chloramphenicol_Acetyltransferase_CAT
Gylcopeptide Resistance
vanH / Dala_Dala_lig_C / DalaDala / vanT / vanY / Dala_Dala_lig_N / vanW
Gene Modulating Resistance
vanR / vanS
Transpeptidase
Transpeptidase
Betalactam Resistance Modulatory Genes
blaI / mecR1
Beta-Lactamase Unclassified
Beta-lactamase / Beta-lactamase2
Beta-Lactamase Class A
PC1 / ClassA
Beta-Lactamase Class B
Lactamase_B / SubclassB3 / Lactamase_B_2 / ClassB
Beta-Lactamase Class C
Beta-Lactamase Class D
Resistant DNA Topoisomerase
Fluoroquinolone Resistant DNA Topoisomerase
Adenylyltransferase
rRNA Methyltransferase
Phosphotransferase
APH3'' / APH3 / APH3'
Nucleotidyltransferase
ANT3 / ANT6 /ANT9
Acetyltransferase
Acetyltransf_1 / Acetyltransf_7 / APH / AAC6-II / Acetyltransf_3 / AAC6-I
Dihydropteroate Synthase
Dihydropteroate
Gene Modulating Resistance
soxR / MarR_2 / baeR / MarR / HTH_AraC / SoxR / baeS
TE Inactivator
TE_Inactivator
Aminotransferase
Aminotran_1_2 / Aminotran_4
Multidrug And Toxic Compound Extrusion Transporter
MATE_efflux
Resistance Nodulation Cell Division Efflux Pump
RND Antibiotic Efflux Pump / RND_mfp / ACR_tran
Small Multidrug Sesistance Antibiotic Efflux Pump
Small_Multi_Drug_Res / emrE
Gene Modulating Antibiotic Efflux Regulating AdeABC
adeR / adeS
Major Facilitator Superfamily Efflux Pump
MFS_1 / emrB / EmrB / MFS Antibiotic Efflux Pump / norA / MFS_3 / Bcr_Cfla
ATP-binding Cassette Efflux Pump
msbA / drrA / macB / ABC_tran / ABC Antibiotic Efflux Pump / macA / ABC2_membrane
